# Transcriptomic and metabolomic analyses reveal the antifungal mechanism of the compound phenazine-1-carboxamide on *Rhizoctonia solani* AG1IA

**DOI:** 10.3389/fpls.2022.1041733

**Published:** 2022-11-22

**Authors:** Ya Zhang, Qiufeng Li, Chong Wang, Shuangqing Liu

**Affiliations:** ^1^ College of Plant Protection, Hunan Agricultural University, Changsha, China; ^2^ College of Bioscience and Biotechnology, Hunan Agricultural University, Changsha, China

**Keywords:** phenazine-1-carboxamide, *Rhizoctonia solani*, transcriptome, differential genes, metabolome, differential metabolites, combined analysis

## Abstract

To explore the molecular mechanisms of the antifungal compound phenazine-1-carboxamide (PCN) inhibits *Rhizoctonia solani* and discover potential targets of action, we performed an integrated analysis of transcriptome and metabolome in *R. solani* mycelium by whether PCN treating or not. A total of 511 differentially expressed genes (DEGs) were identified between the PCN treatment and control groups. The fluorescence-based quantitative PCR (qPCR) got the accordant results of the gene expression trends for ten randomly selected DEGs. The Gene Ontology (GO) enrichment analysis revealed that fatty acid metabolic process, fatty acid oxidation, and lipid oxidation were among the most enriched in the biological process category, while integral component of membrane, plasma membrane, and extracellular region were among the most enriched in the cellular component category and oxidoreductase activity, cofactor binding, and coenzyme binding were among the most enriched in the molecular function category. KEGG enrichment analysis revealed the most prominently enriched metabolic pathways included ATP-binding cassette (ABC) transporters, nitrogen metabolism, aminobenzoate degradation. The DEGs related functions of cellular structures, cell membrane functions, cellular nutrition, vacuole-mitochondrion membrane contact site and ATPase activity, pH, anti-oxidation, were downregulated. A total of 466 differential metabolites were found between the PCN treatment and control groups after PCN treatment. KEGG enrichment found purine, arachidonic acid, and phenylpropanoid biosynthesis pathways were mainly affected. Further results proved PCN decreased the mycelial biomass and protein content of *R. solani*, and superoxide dismutase (SOD) activity reduced while peroxidase (POD) and cytochrome P450 activities increased. The molecule docking indicted that NADPH nitrite reductase, ATP-binding cassette transporter, alpha/beta hydrolase family domain-containing protein, and NADPH–cytochrome P450 reductase maybe the particular target of PCN. In conclusion, the mechanisms *via* which PCN inhibits *R. solani* AG1IA may be related to cell wall damage, cell membrane impairment, intracellular nutrient imbalance, disturbed antioxidant system, and altered intracellular pH, which laid foundation for the further new compound designing to improve antifungal efficacy.

## Introduction

Rice is an important staple food crop mainly cultivated in Asia, southern Europe, tropical America, and parts of Africa. Limited by geography and climatic conditions, the planting area of rice is lower than that of wheat, but it sustains more of the human population (Kakar et al., 2019; de Barreda et al., 2021; Ibrahim et al., 2021). The stable production and high yield of rice influences national food security. As a country’s population continues to grow, increasingly higher yields of rice are needed to meet food demands. Otherwise, the contrary can lead to imbalanced supply and demand, resulting in food shortage, further impacting a country’s long-term stability and prosperity (Huang, 2021; Shen et al., 2021). The growth process of rice is often modulated by various factors, such as fertilizers, soil texture, planting density, climate, plant diseases, and insect pests. These external factors are stochastically amplified from time to time, severely affecting yield and the requirement for a bumper harvest of rice to meet human consumption demands (Han et al., 2018; Wu et al., 2021; Guo et al., 2021; Lu et al., 2021; Wang et al., 2021).

Rice sheath blight is one of the most serious diseases caused by *Rhizoctonia solani* AG1IA and generally occurs in every paddy rice field. Due to its soil-borne characteristics, good resistance of pathogen, high degree of invisibility to surveillance, and a wide host range of this disease, it has been very difficult to eradicate (Keijer et al., 1997; Singh et al., 2016; Feng et al., 2017). Presently, methods for controlling and preventing this disease during production primarily include removing floating sclerotia, reasonable fertilization, adjusting planting density, breeding resistant rice varieties, and chemical application of fungicides, among which validamycin is the most commonly used agent (Jones, 1987; Li et al., 1989; Slaton et al., 2003; Zhang et al., 2009; Chen et al., 2012; Wu et al., 2015; Chen et al., 2016). However, since the introduction of validamycin 30 years ago, the risk of microbial drug resistance has increased as a result of its long-term exclusive utilization in large quantities. The European Union also prohibits the sales of agents including validamycin due to the potential risks of antibiotic resistance on the health of humans, animals, and plants, as well as due to environmental safety concerns. Thus, the limitation of the application of validamycin in Europe poses a threat to the safety of rice production in China (Shen, 1985; Smith et al., 2005). Therefore, it is urgently necessary to explore new ways to control and prevent rice sheath blight.

Our research group previously found that phenazine-1-carboxamide (PCN; CAS: 550-89-0; Molecular formula: C_13_H_9_N_3_O; Molecular weight: 223.23; Melting point: 241°C) inhibited the pathogen *R. solani* AG1IA effectively at a 50% effective concentration (EC_50_) of 9.09 μg·mL^−1^. Its action mechanisms for inhibiting *R. solani* AG1IA were studied preliminarily, but the depth of this investigation was insufficient and was lacking in interpretation at the molecular level, affecting the later development and application of this pesticide (Xiang et al., 2018). Based on this, the present study employed multiple approaches, including transcriptomics, fluorescence-based quantitative PCR (qPCR), metabolomics, physiological and biochemical tests for key indicators, and verification of drug target models. We clarified the changes of key genes and metabolites in *R. solani* AG1IA treated with PCN, studied the correlation between the two, and investigated possible new targets of action of PCN. This study revealed the antifungal mechanisms of the antifungal compound PCN at the molecular level and provides new insights and strategies for the management of rice sheath blight.

## Materials and methods

### Test fungal strain and reagent

The *Rhizoctonia solani* AG1IA strain was provided by the Laboratory of Excavation and Utilization of Biocontrol Resources of Hunan Agricultural University. The antifungal compound PCN at a concentration of 92.03% was produced by the Biopesticide Engineering Center of Hunan Agricultural University.

### Treatment of transcriptome samples

*R. solani* AG1IA was inoculated at the center of potato dextrose agar (PDA: Potato 200 g; Dextrose 20 g; Agar 15 g; H_2_O 1000 mL) medium, and the plates were placed in an incubator at 26 – 28°C for continuous culture of 48 h. Activated strains were prepared before use. PCN was dissolved in trace acetone and mixed with sterile water to prepare a stock solution at a concentration of 400 μg·mL^−1^, which was then added into potato dextrose broth (PDB: Potato 200 g; Dextrose 20 g; H_2_O 1000 mL) medium to prepare the drug-containing culture medium at a final concentration of 40 μg·mL^−1^. Drug-free PDB medium was used as a control, and each treatment was repeated three times, which were named TM1, TM2, TM3, CK1, CK2, and CK3, respectively. Cultures were placed in a shaker at 26 – 28°C with shaking of 48 h at 120 r·min^−1^ (Xiang et al., 2018). The mycelium were then collected and stored in a −80°C freezer for RNA isolation.

### RNA isolation of *R. solani* AG1IA

Total RNA from *R. solani* AG1IA was extracted using the TRIzol method. The degree of RNA degradation and presence of contamination were analyzed by agarose gel electrophoresis. RNA purity was examined by Nanodrop (Thermo Fisher Scientific, Waltham, MA, USA). RNA concentration was quantified using the Qubit RNA quantification kit, and RNA integrity was determined using an Agilent 2100 BioAnalyzer (Zhang et al., 2022). After RNA samples passed quality inspection, oligo(dT) coupled to magnetic beads were used to enrich for mRNAs. Subsequently, fragmentation buffer was added to digest these mRNAs into short fragments. First strand complementary DNA (cDNA) was synthesized using mRNA as a template and random hexamers as primers. Second strand cDNA chain was then synthesized by adding buffer, dNTPs, DNA polymerase I, and RNase H. AMPure XP beads were then utilized to purify double-stranded cDNA (dscDNA). The purified dscDNA was end repaired, A tailed, and ligated with sequencing adaptors, which were then size selected using AMPure XP beads. Finally, PCR amplification was performed, and PCR products were purified by AMPure XP beads to derive final libraries. After the completion of library construction, a Qubit 2.0 was utilized for initial concentration determination. The libraries were diluted to 1 ng/μL, and the inserted fragment sizes of these libraries were then determined using an Agilent 2100 BioAnalyzer. Once the inserted fragments met specifications, the effective concentration of each library was accurately quantified using qPCR (effective concentration of library > 2 nM) to ensure library quality for sequencing.

### High-throughput sequencing and data analysis

For the libraries passing quality inspection, pooling of different libraries for loading on a flow cell was performed according to the effective concentrations and targeted sequencing raw data amount required. After clustering on a cBOT, paired-end (PE) sequencing was conducted using an Illumina high-throughput sequencing platform (HiSeq/MiSeq). After acquiring raw reads, we first assessed raw reads (paired-end reads) derived from sequencing, and when reference sequences or a reference genome of related species existed, bioinformatics analysis was performed using the following procedures. For the raw data quality control and evaluation, raw sequencing data was filtered and assessed for quality using fastp (Chen et al., 2018) software. For ribosomal RNA removal, we compared the above sequences passing quality inspection with the ribosomal RNA sequences in the Rfam database, and the unaligned reads were retained for subsequent analysis. For reference genome comparison, we employed Hisat2 (Sirén et al., 2014; Kim et al., 2015) to align the filtered reads to a reference genome to derive SAM/BAM files for each sample comparison. For read count quantification, RESM was used to calculate the read counts for each sample. For differential gene analysis, after transcript quantification based on reads count, edgeR v3.28.1 (adjust method = “BH”, p.value/FDR threshold = 0.05, |log2(FC) | > 1; Robinson et al., 2010; Anders and Huber, 2010) was used to analyze differential expression, thereby identifying genes with differences in expression between the treated and control samples. For an enrichment analysis of differential genes, for significantly different genes, clusterProfiler (Yu et al., 2012) was used to perform enrichment analysis of the functions and pathways of the input differential genes. For novel transcript prediction, StringTie (Pertea et al., 2015; Pertea et al., 2016) was used to assemble transcripts, compared to a reference genome (ID: 16395), and identify novel transcripts. rMATS software (Wang et al., 2010) was used to analyze alternative splicing. ANNOVAR (Yang and Wang, 2015) was used for SNP and INDEL analysis and annotation.

### Quantitative real-time PCR validation of DEGs

*R. solani* AG1IA culture and total RNA isolation were performed using the methods described in treatment of transcriptome samples and RNA isolation of *R. solani* AG1IA. Reverse transcription was performed with 100 ng of total RNA using HiScript Q Select RT SuperMix for qPCR (+gDNA wiper) (Vazyme). Using reverse transcribed products as templates, quantitative real-time PCR (qRT-PCR) was then performed using ChamQTM Universal SYBR^®^ qPCR Master Mix (Vazyme) on a qTOWER 2.2 instrument (Germany). Gene-specific primers were designed using Primer Premier 6.0 software. Using 18S rRNA as an endogenous reference, ten genes that differed significantly were randomly chosen for primer design and qRT-PCR validation. The tested genes and primers are displayed in **Supplementary Table 1**.

### Treatment and extraction of metabolome samples

*R. solani* AG1IA was treated using the methods described in treatment of transcriptome samples, and the collected mycelium were stored in a freezer at −80°C before testing. For the extraction of metabolites, the samples were taken out from the −80°C freezer and thawed on ice to a state capable of being physically broken up. The samples were minced and mixed well, and multipoint sampling was performed followed by weighing 20 mg (+1 mg) into centrifuge tubes with corresponding numbers. Steel balls were added to each tube using tweezers, and a ball mill (30 HZ) was used to homogenize each sample for 20 s. The samples were centrifuged by spinning at 3000 r/min for 30 s at 4°C. After centrifugation, 400 μL of a 70% methanol water internal standard extraction solution was added. The samples were oscillated at 1500 r/min for 5 min and then incubated on ice for 15 min. Keeping the samples at 4°C, the samples were then centrifuged at 12000 r/min for 10 min, and 300 μL of supernatant was transferred to another centrifuge tube with corresponding numbers. The samples were stored in a −20°C freezer for 30 min, followed by centrifuging again at 12000 r/min for 3 min at 4°C. A volume of 200 μL supernatant was finally transferred into corresponding sampling lined vials and subjected to instrumental analysis.

### Metabolite testing conditions

The data acquisition instrument systems mainly included an ultra-performance liquid chromatograph (UPLC) (ExionLC AD, https://sciex.com.cn/) and a tandem mass spectrometer (MS/MS) (QTRAPB, https://sciex.com/). Chromatographic acquisition conditions using the T3 method were as follows. Chromatographic column, Waters ACQUITY UPLCHSS T3 C18 1.8 μm, 2.1 mm × 100 mm; Mobile phase, Phase A was ultrapure water and Phase B was acetonitrile; Elution gradient, water/acetonitrile was 95:5 (V/V) at 0 min, 10:90 (V/V) at 11.0 min, 10:90 (V/V) at 12.0 min, 95:5 (V/V) at 12.1 min, and 95:5 (V/V) at 14.0 min; Flow velocity, 0.35 m/min; Column temperature, 40°C; and injection volume, 2 μl. Mass spectrum acquisition conditions using the T3 method were as follows: Electrospray ionization (ESI) source temperature, 500°C; MS voltages, 5500 V (positive) and −4500 V (negative); ion source Gas I (GSI), 50 psi and Gas II (GSII), 50 psi; curtain gas (CUR), 25 psi; collision-activated dissociation (CAD) parameter, high. For triple quadrupole (Qtrap) assays, scan detection was performed for each ion pair according to their optimized declustering potentials (DP) and collision energies (CE).

### Combined transcriptome and metabolome analysis

Using our transcriptome and metabolome data, based on methods including annotation and enrichment of the Kyoto Encyclopedia of Genes and Genomes (KEGG) pathways, Pearson’s correlation coefficients (The Pearson’s correlation coefficients were calculated for the transcriptome and metabolome data by cor() function in R package. The correlations that corresponded to a coefficient of P value < 0.05 and R2>0.8 were selected. Finally, Cytoscape (3.6.1) software was employed to visualize the relationships between the transcriptome and metabolome), and dimensionality reduction model building for association relationship determination, function analysis (KEGG pathways and KEGG enrichment), expression trend analysis (nine-quadrant), and expression level association analyses (correlation analysis and canonical correlation network) were performed to clarify the relationships between biological processes and phenotypes.

### Effect of PCN on the biomass of *R. solani* AG1IA

*R. solani* AG1IA were inoculated at the center of PDA medium and cultured in an incubator at 26 – 28°C in the dark for 48 h. A fungal plug of *R. solani* AG1IA with a diameter of 5 mm was punched from each plate and inoculated into 0, 10 μg·mL^−1^, and 40 μg·mL^−1^ drug-containing PDB medium, with each treatment repeated three times. These culture were then grown in a shaker at a speed of 120 r·min^−1^ for 48 h at 26 – 28°C. The culture medium was filtered out and each agar block was taken out with tweezers. Each fungal ball of mycelium was rinsed with sterile distilled water three times, dried in a 60°C oven for 24 h, and weighed.

### Effect of PCN on the protein content of *R. solani* AG1IA

*R. solani* AG1IA were inoculated at the center of PDA medium and cultured in an incubator at 26 – 28°C in the dark for 48 h. A 5 mm fungal plug was punched from each plate and inoculated into 0, 10 μg·mL^−1^, 40 μg·mL^−1^ drug-containing PDB medium. Each treatment was repeated three times. These cultures were grown in a constant temperature incubator at 26 –28°C for 48 h. After the mycelium was taken out using tweezers, the residual medium on mycelium was drained with a Büchner funnel. After rinsing with phosphate buffer two times, 1 g of dried mycelium was added to a mortar along with 2 mL of phosphate buffer and 1 g of quartz sand. The mortar was placed on ice for grinding until it become homogeneous, which was then transferred to centrifuge tubes. Phosphate buffer was added to a total volume of 10 mL, these tubes were then centrifuged at 13000 r·min^−1^ for 5 min, and the supernatant was removed and preserved at −20°C. A standard curve was created using BSA. Distilled water was added to 0.5 mL of each sample solution to a final volume of 1 mL, and 5 mL of Coomassie brilliant blue G-250 reagent was added. Phosphate buffer was used as a blank control. Optical density (OD) values were measured at a wavelength of 660 nm, and the soluble protein content in each sample was calculated (Bradford, 1976).

### Effect of PCN on the extracellular pH of *R. solani* AG1IA

*R. solani* AG1IA were inoculated at the center of PDA medium and cultured in an incubator at 26 –28°C in the dark for 48 h. A fungal plug with a diameter of 5 mm was punched from each plate and inoculated into 0, 10 μg·mL^−1^, 40 μg·mL^−1^ drug-containing PDB medium. Each treatment was repeated three times. These cultures were grown in a shaker at 120 r·min^−1^ for 48 h at 26 – 28°C. A volume of 10 mL of fungal culture was aspirated and centrifuged at 10000×g for 3 min. The supernatant after centrifugation was collected and tested for the fungal extracellular pH value using an AZ8601 portable pH meter (Sun, 2018).

### Effect of PCN on the activities of key enzymes

*R. solani* AG1IA cultures were treated using the methods described in Section 2.9. A Superoxide Dismutase (SOD) Assay Kit (A001-3, Nanjing Jiancheng Bioengineering Institute) was utilized to test SOD activities. A Peroxidase (POD) Assay Kit (Cat#BC0090, Beijing Solarbio Science & Technology Co., Ltd.) was utilized to test POD activities. A Cytochrome P450 Enzyme Assay Kit (A114608, Shanghai Fusheng Industry Co., Ltd.) was utilized to test P450 function.

### Receptor homology modeling and molecular docking of PCN

Based on the results of our transcriptome and metabolome integrated analysis, four likely receptor genes with significant differences and downregulated expression were selected from co-enriched pathways. The Swiss modeling and Pyhre2 methods were used to construct protein models, based on which the binding mechanisms between PCN and targets were investigated (Zhang et al., 2018; Fang et al., 2020).

### Data analysis

All of the data was processed using Excel, and for statistical analysis, DPS v6.55 was utilized to compare data using the Duncan’s new multiple range test method.

## Results

### Sequencing data quality analysis

The total reads of TM1, TM2, TM3, and CK1, CK2, CK3, were 33161030, 20040436, 22001342, 26445898, 28483697, 20342574, respectively (**Supplementary Table 2** **)**. The Q20% (percentage of base mass greater than Phred score Q20 to total base ratio) obtained was 98.55%, 98.53%, 98.60%, 98.63%, 98.53%, and 98.11%, respectively. The Q30 (percentage of base mass greater than Phred score Q30 to total base ratio) was 94.69%, 94.65%, 94.85%, 94.93%, 94.64%, and 93.32%, respectively. These results indicate that the data acquired from sequencing were of good quality.

### Gene expression characteristics and differential level analysis

**Figure 1:**
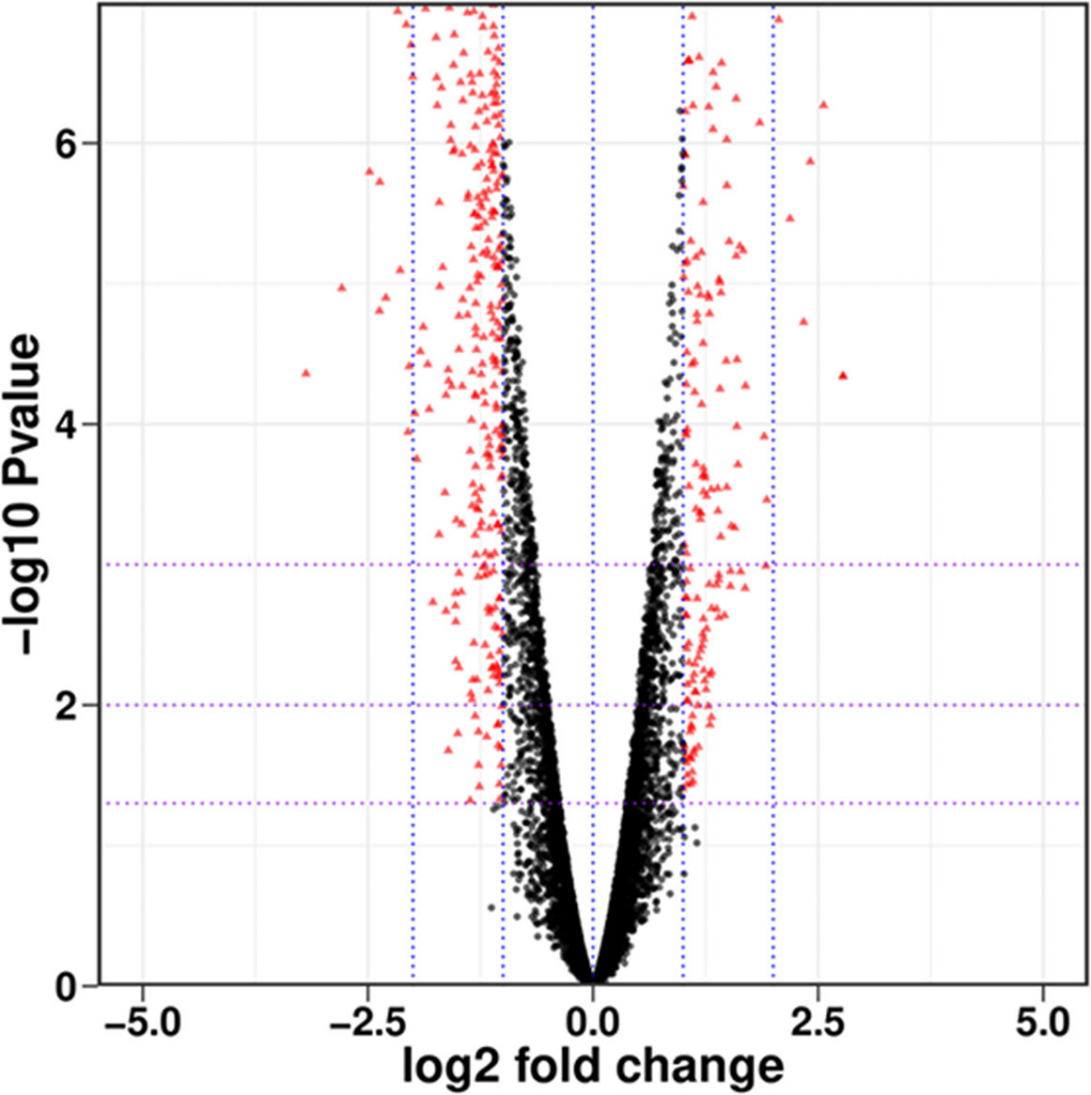
Overall distribution of differentially expressed genes (DEGs). Each dot represents a gene, with red indicating significantly different genes and black indicating genes with no significant difference.

As shown in **Figure 1**, there were a total of 7066 genes between PCN-treated *R. solani* AG1IA and control, including 3650 upregulated genes and 3416 downregulated genes. Among all genes, there were many genes with small absolute values of expression changes and significant differences. There were in total 511 differentially expressed genes (DEGs) whose absolute values of Log_2_ fold changes of expression were greater than 1 with significant differences, including 164 upregulated genes and 347 downregulated genes.

### GO functional clustering analysis

**Figure 2:**
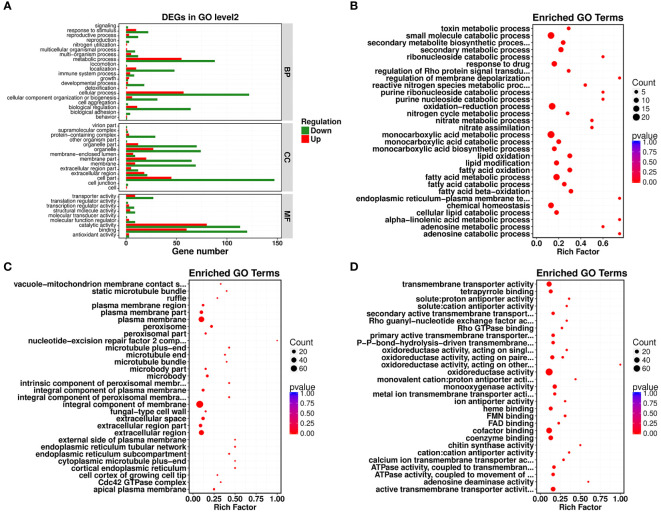
Gene Ontology (GO) enrichment analysis of PCN-related DEGs. **(A)** GO functional classification; **(B)** biological process (BP); **(C)** cellular component (CC); **(D)** molecular function (MF).

Gene Ontology (GO) functional enrichment analysis revealed that significantly different genes could be primarily divided into three basic categories: biological processes (BP), cellular components (CC), and molecular functions (MF). In the biological process category, most DEGs were associated with cellular process, metabolic process, biological regulation, and localization, with 179, 143, 75, and 58 DEGs included, which accounted for 2.53%, 2.02%, 1.06%, and 0.82% of the total number of DEGs, respectively (**Figure 2A**). In the cellular component category, cell part, organelle, organelle part, membrane part, and membrane contained the largest numbers of DEGs. Their corresponding DEG counts were 192, 101, 82, 85, and 78, accounting for 2.72%, 1.43%, 1.16%, 1.20%, and 1.10% of the total number of DEGs, respectively (**Figure 2A**). In the molecular function category, most DEGs discovered were associated with catalytic activity, binding, and transporter activity, with 193, 180, and 36 DEGs, accounting for 2.73%, 2.55%, and 0.51% of the total number of DEGs, respectively (**Figure 2A**). In all of the most influential subcategories, such as cellular process, metabolic process, biological regulation, localization, cell part, organelle, organelle part, membrane part, membrane, catalytic activity, binding, and transporter activity, the numbers of downregulated genes were all significantly higher than the numbers of upregulated genes. This suggested that targeted genes and functions of *R. solani* AG1IA were inhibited by PCN, thereby preventing the growth and proliferation of this pathogenic fungi.

Under the biological process classification, the most significantly enriched GO terms included fatty acid metabolic process, fatty acid oxidation, lipid oxidation, oxidation-reduction process, monocarboxylic acid metabolic process, and small molecule catabolic process. There were far more downregulated genes than upregulated genes regulating these functions, which indicated again that PCN repressed gene expression and impaired the biological function of this pathogenic fungi (**Figure 2B**). Under the cellular component classification, the most significantly enriched GO terms included integral component of membrane, plasma membrane, extracellular region, extracellular space, peroxisome, microbody, and apical plasma membrane, among which there were 60 downregulated DEGs associated with the function integral component of membrane (**Figure 2C**). Under the molecular function classification, the most significantly enriched GO terms included oxidoreductase activity, cofactor binding, coenzyme binding, active transmembrane transporter activity, chitin synthase activity, heme binding, and tetrapyrrole binding, among which there were 68 downregulated DEGs associated with oxidoreductase activity and cofactor binding (**Figure 2D**).

### KEGG enrichment analysis

**Figure 3:**
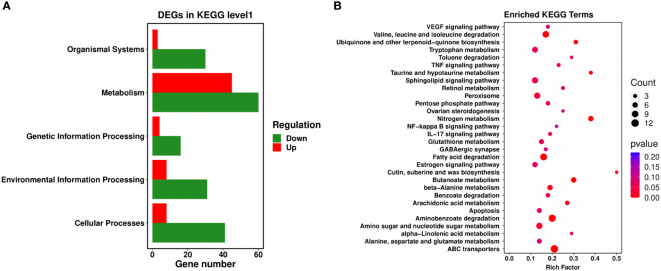
KEGG enrichment analysis of PCN-related DEGs. **(A)** level 1 metabolic pathways; **(B)** level 2 metabolic pathways.

Next, a comparison between our DEGs and the KEGG database was performed, and the significantly enriched metabolic pathways within our set of DEGs were identified to analyze the physiological processes these DEGs participated in. Our results revealed 22 metabolic pathways with a corrected *P*-value ≤ 0.05 as a threshold, which mainly included ATP-binding cassette (ABC) transporters, nitrogen metabolism, aminobenzoate degradation, fatty acid degradation, valine, leucine and isoleucine degradation, arachidonic acid metabolism, pentose phosphate pathway, and the sphingolipid signaling pathway. Among them, the most prominent enrichments were in metabolic pathways, including ABC transporters, nitrogen metabolism, and aminobenzoate degradation (**Figures 3A, B**).

### qRT-PCR validation

**Figure 4:**
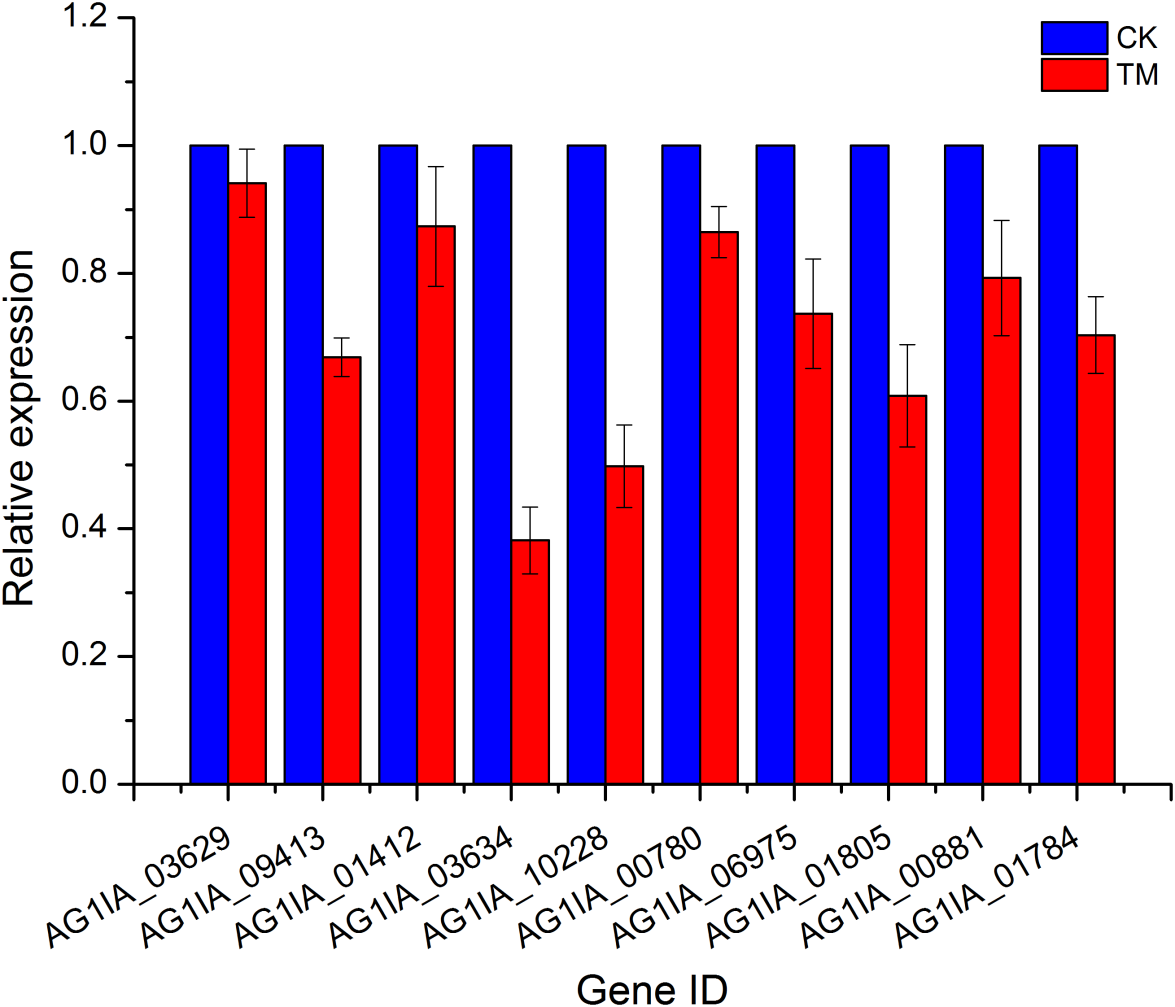
qRT-PCR validation of PCN-related DEGs.

Ten genes were then randomly selected from the above transcriptome data for qRT-PCR validation, and these qRT-PCR validation results using 18S rRNA as an endogenous reference were consistent with the expression trends from our transcriptome data (**Figure 4**), again validating the high sequencing quality of our transcriptome experiments.

### Genes related to cell wall

In PCN-treated *R. solani* AG1IA, the chitin synthase activity function displayed reduced expression in genes encoding glycosyltransferase family 2 protein (AG1IA_09260 and AG1IA_07549), chitin synthase 6 (AG1IA_07339), and chitin synthase G (AG1IA_04366), while upregulated genes were not found. In the fungal-type cell wall function, two key genes, glycoside hydrolase family 16 protein (AG1IA_09291) and alpha-1,3 glucan synthase (AG1IA_00910), were downregulated, while no upregulated genes were found (**Supplementary Table 3**). We speculated that the cell wall of *R. solani* AG1IA was impacted by the actions of PCN, leading to changes in its composition and structure.

### Genes related to cell membrane

Fatty acids and lipids are the principal components of biological membranes, and are components of cell membranes, mitochondrial membranes, and plasma membranes. They play pivotal roles in most membranes and are involved in phospholipid synthesis. In PCN-treated *R. solani* AG1IA, 10 key genes in the fatty acid metabolic process pathway had decreased expression, which included ATP-binding cassette transporter (AG1IA_00780), PEX11 domain-containing protein (AG1IA_09459), acetyl-CoA acetyl transferase (AG1IA_01871), linoleate diol synthase (AG1IA_06477), putative acyl-coenzyme a oxidase 3.2, peroxisomal (AG1IA_09252), fatty acid oxygenase (AG1IA_01703), peroxysomal citrate synthase (AG1IA_05287), alpha/beta hydrolase family domain-containing protein (AG1IA_02292), hydroxysteroid dehydrogenase (AG1IA_05255), and carnitine acetyl transferase (AG1IA_08166). In the arachidonic acid metabolic pathway, there were three genes, alpha/beta hydrolase family domain-containing protein (AG1IA_02292), linoleate diol synthase (AG1IA_06477), and fatty acid oxygenase (AG1IA_01703), with downregulated expression (**Supplementary Table 3**). These genes play crucial roles as phospholipid-binding structural lipids, which suggested that PCN may damage the cell membrane structure of this pathogenic fungus.

In the fatty acid metabolic process pathway, there were five upregulated genes, including phenylacetyl-CoA ligase (AG1IA_09040), CFS1-like protein (AG1IA_01990), enoyl-CoA hydratase (AG1IA_02110), enoyl-CoA hydratase/isomerase family protein (AG1IA_04388), and hypothetical protein (AG1IA_05814). In the fatty acid oxidation pathway, two genes, enoyl-CoA hydratase/isomerase family protein (AG1IA_04388) and enoyl-CoA hydratase (AG1IA_02110), were upregulated. In terms of plasma membrane function, seven genes, including TPR_1 domain-containing protein (AG1IA_01398), major facilitator superfamily protein (AG1IA_03838), glycosyl hydrolase catalytic core domain-containing protein (AG1IA_04180), and hypothetical protein (AG1IA_05884), had increased expression. These results suggested that this fungus augments the synthesis of its cell membrane after PCN treatment in order to restore the cell membrane destroyed by PCN.

### Genes related to cellular nutrition

Nitrogen is the basic building constituent for protein, DNA, and RNA in cells, and altered intracellular metabolism of nitrogen may imply the risk of apoptosis. The expression of six genes involved in the function of nitrogen metabolism was downregulated after PCN treatment, which included NADPH nitrite reductase (AG1IA_09410), nitrate transporter (AG1IA_09412), 2-nitropropane dioxygenase (AG1IA_06435), NADPH nitrite reductase (AG1IA_09411), nitrate reductase (AG1IA_09413), and 2-nitropropane dioxygenase (AG1IA_01412) (**Supplementary Table 3**). This suggested that PCN may cause nutritional dysfunction in this pathogen. Six genes involved in the aminobenzoate degradation function, including FAD binding domain-containing protein (AG1IA_01905), cytochrome P450 monooxygenase (AG1IA_04867), enoyl-CoA hydratase/isomerase family protein (AG1IA_04388), cytochrome P450 monooxygenase (AG1IA_09272), FAD binding domain-containing protein (AG1IA_04881), and cytochrome P450 monooxygenase (AG1IA_00318), had upregulated expression. In the function of valine, leucine and isoleucine degradation, six genes, including zinc-binding dehydrogenase domain-containing protein (AG1IA_03872), enoyl-CoA hydratase/isomerase family protein (AG1IA_04388), enoyl-CoA hydratase (AG1IA_02110), alcohol dehydrogenase groES-like domain-containing protein (AG1IA_05942), coenzyme A transferase domain-containing protein (AG1IA_08405), and branched-chain alpha-keto acid dehydrogenase E1-alpha subunit (AG1IA_01275), showed increased expression. Additionally, two genes involved in the tryptophan metabolism pathway had upregulated expression. These results suggested that this fungus may enhance its nutrient synthesis after PCN treatment in order to renovate the nutrition compromised by PCN treatment.

### Genes related to energy metabolism

The expression of two genes, P5-ATPase domain-containing protein (AG1IA_08254) and calcium permease (AG1IA_03168), which were involved in the function of vacuole-mitochondrion membrane contact site, was downregulated (**Supplementary Table 3**). Within the function of ATPase activity (coupled to transmembrane movement of substances and coupled to movement of substances), 11 target genes were downregulated, including calcium-transporting ATPase 3 (AG1IA_02536), P5-ATPase domain-containing protein (AG1IA_08254), cation-transporting atpase fungi (AG1IA_05862), plasma membrane H(+)-ATPase 1 (AG1IA_04742), multidrug resistance-associated ABC transporter (AG1IA_06082), ATP-binding cassette transporter (AG1IA_00780), multidrug resistance protein 4 (AG1IA_06975), ABC transporter domain-containing protein (AG1IA_03597), hypothetical protein (AG1IA_03328), ABC multidrug transporter, putative (AG1IA_01805), and ABC transporter CDR4 (AG1IA_01784). However, four genes involved in this function, including ABC transporter (AG1IA_08015), ABC transporter (AG1IA_06165), ABC drug exporter AtrF (AG1IA_09306), and ABC transporter (AG1IA_02225), were upregulated. Thus, PCN may interfere with the energy metabolism of this pathogenic fungus.

### Genes related to pH

The gene expression of adenosine deaminase (AG1IA_03192) within the function of solute:proton antiporter activity was downregulated, while two genes, including adenosine deaminase (AG1IA_03187) and adenosine deaminase (AG1IA_03188), showed upregulated expression (**Supplementary Table 3**). These results indicated that PCN may also alter the cellular pH environment of this pathogenic fungus.

### Genes related to antioxidative stress

The gene expression of ATP-binding cassette transporter (AG1IA_00780), peroxisomal membrane protein (AG1IA_04529), peroxysomal citrate synthase (AG1IA_05287), alpha/beta hydrolase family domain-containing protein (AG1IA_02292), PEX11 domain-containing protein (AG1IA_09459), hydroxysteroid dehydrogenase (AG1IA_05255), and carnitine acetyl transferase (AG1IA_08166), which have been shown to be involved in peroxisome function, were downregulated (**Supplementary Table 3**). For the function of oxidoreductase activity, 35 genes had downregulated expression, which included aldehyde dehydrogenase (AG1IA_03762), cytochrome P450 (AG1IA_03903), laccase precursor (AG1IA_08835), NADPH–cytochrome P450 reductase (AG1IA_03629), 2-nitropropane dioxygenase (AG1IA_01412), and cytochrome P450 monooxygenase pc-3 (AG1IA_09085). In terms of the function monooxygenase activity, 13 genes including NADPH–cytochrome P450 reductase (AG1IA_03634), cytochrome P450 (AG1IA_08511), cytochrome P450 monooxygenase pc-3 (AG1IA_07677), tyrosinase domain-containing protein (AG1IA_07059), cytochrome P450 (AG1IA_03903), P450 family fatty acid hydroxylase (AG1IA_09807), Rieske [2Fe-2S] domain-containing protein (AG1IA_08914), 2-nitropropane dioxygenase (AG1IA_06435), NADPH–cytochrome P450 reductase (AG1IA_03629), hypothetical protein (AG1IA_08606), 2-nitropropane dioxygenase (AG1IA_01412), cytochrome P450 monooxygenase pc-3 (AG1IA_09085), and cytochrome P450 domain-containing protein (AG1IA_10228) showed downregulated expression. In contrast, two genes phenylacetyl-CoA ligase (AG1IA_09040) and HIT domain-containing protein (AG1IA_08976) in the peroxisome function had upregulated expression. The expression of 35 genes was upregulated in the function oxidoreductase activity, which included NADH:flavin oxidoreductase/NADH oxidase (AG1IA_01788), hypothetical protein (AG1IA_00127), salicylate 1-monooxygenase (AG1IA_08900), isoamyl alcohol oxidase, putative (AG1IA_09626), Glutaredoxin domain-containing protein (AG1IA_02038), monooxygenase (AG1IA_08597), and iron/ascorbate oxidoreductase (AG1IA_03269). These results indicated that PCN could impair the antioxidative activities of this pathogen.

### Genes related to pathogenicity

The pathogenicity was weakened in this fungus when it was treated with PCN, which resulted in significantly downregulated expression of glycoside hydrolase family 61 protein (AG1IA_06330), glycoside hydrolase family 16 protein (AG1IA_09291), and glycoside hydrolase family 79 protein (AG1IA_01834) genes, suggesting that they might be potential acting targets (**Supplementary Table 3**). In contrast, the gene expression of glycoside hydrolase family 3 protein (AG1IA_00256), glycoside hydrolase family 16 protein (AG1IA_02785), and glycoside hydrolase family 15 protein (AG1IA_02474) was upregulated, whose expression may be enhanced by PCN to resist the damaging effects of PCN.

### Effect of PCN on the metabolome of *R. solani* AG1IA

**Figure 5:**
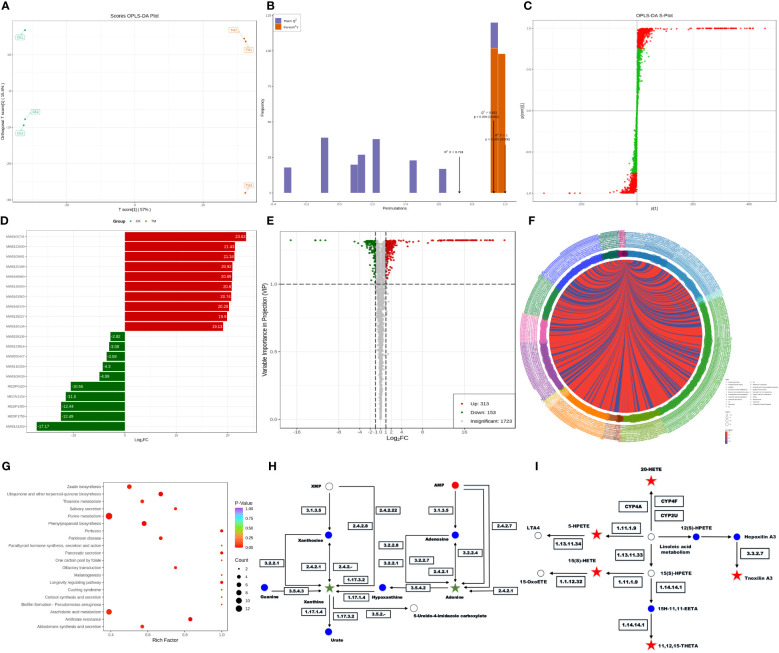
Effect of the antifungal compound PCN on the *R. solani* AG1IA metabolome. **(A)** OPLS-DA scoring result; **(B)** OPLS-DA model validation outcome; **(C)** S-plot of OPLS-DA; **(D)** bar graph of differential metabolites; **(E)** volcano plot of differential metabolites; **(F)** chord diagram of differential metabolites; **(G)** KEGG enrichment plot of differential metabolites; **(H)** KEGG pathway diagram of purine differential metabolites; **(I)** KEGG pathway diagram of arachidonic acid key differential metabolites.

The metabolome is the downstream products of the transcriptome and proteome and is tightly coupled to the phenotype of biological systems, which can directly reflect the terminal and phenotypic information of an organism (Atanasova-Penichon et al., 2018). Studies of the metabolome are promising for accelerating an understanding of unknown genes through testing the metabolic status of pathogens. Principal component analysis (PCA) among unsupervised analysis methods is a common classification method in metabolomics, but the differences within groups are often greater than the differences between groups, and the results from such analyses are usually unreliable. Thus, it is necessary to employ supervised analysis method for group separation. In the key parameters of the orthogonal projections to latent structures discriminant analysis (OPLS-DA), R^2^X, R^2^Y, and Q^2^ were all greater than 0.5, indicating our model had high accuracy and predictive capacity. An S-plot revealed the differential metabolites related to groups, with the absolute values of P[1] and P(corr)[1] greater than 0.05 and 0.5, respectively. After a *t*-test, the metabolites with *P* < 0.05 were considered differential metabolites. As shown in **Figure 5A**, OPLS-DA significantly separated the treatment group and control group with small within-group differences, demonstrating a certain predictive capacity of this model. Further verification showed that the main parameters of OPLS-DA were R^2^X = 0.724, R^2^Y = 1, and Q^2^ = 0.931, demonstrating that this model had good predictive capacity and accuracy (**Figure 5B**). The corresponding S-plot displays the metabolites related to grouping in the two samples (**Figure 5C**). There were in total 466 differential metabolites in the PCN-treated *R. solani* AG1IA, among which 313 metabolites were upregulated and 153 metabolites were downregulated (**Supplementary Table 4**). **Figure 5D** shows that the upregulated metabolite with the most significant difference was arginyl-tryptophan (MW0105731), while the downregulated metabolite with the most significant difference was 13,14-dihydro PGF2 (MW0141353), and the absolute value of the differential fold of the former was 1.35 times that of the latter. Among all differential metabolites, the top ten most significantly upregulated metabolites had evidently higher differential folds than the top ten most significantly downregulated metabolites. These results indicated that the differential metabolites mainly exert resistance function under the influence of PCN. As shown in **Figure 5E**, among all metabolites, there was a much larger number of differential metabolites that were significantly upregulated compared to those downregulated, and the upregulated had more relative metabolite content compared to the downregulated, which also implied that the differential metabolites may exert resistant effects. As is shown in **Figure 5F**, metabolites including tryptamines, cholines, and pigments were the most crucial substances, which play important roles in the tryptophan metabolism pathway. Amino acids and their metabolites were second, which were followed sequentially by nucleic acid metabolites and their derivatives, organic acid metabolites, fatty acid metabolites, and benzene metabolites. There were positive correlations between these metabolites, indicating their possible same functions. At the same time, some metabolites were negatively correlated, suggesting non-overlapping functions between these metabolites. Moreover, N-methyltryptamine (MEDP0123) was the most critical metabolite, which was positively corelated with most differential metabolites while negatively correlated with a minority of differential metabolites, indicating a great influence of PCN on this metabolite. As shown in **Figure 5G**, purine metabolism was the pathway most affected by PCN, arachidonic acid metabolism was the second, and phenylpropanoid biosynthesis was the third, followed by ubiquinone and other terpenoid-quinone biosynthesis, thiamine metabolism, zeatin biosynthesis, melanogenesis, and biofilm formation - pseudomonas aeruginosa, while other metabolic pathways were relatively less affected by PCN treatment. In the phenylpropanoid biosynthesis metabolic pathway, umbelliferone and scopolin were affected greatly. Moreover, in the metabolic pathways of thiamine metabolism and melanogenesis, tyrosine was impacted prominently and was significantly downregulated. In addition, the significantly downregulated O-β-D-glucosyl-*trans*-zeatin in the zeatin biosynthesis metabolic pathway had a considerable influence on the metabolome. **Figure 5H** shows that in the purine metabolism pathway, the activity changes of seven enzymes including guanine deaminase, purine nucleosidase, purine-nucleoside phosphorylase, and xanthine dehydrogenase resulted in significant downregulated xanthine, leading to a dysregulation of purine nucleotides. Secondly, the activity changes of six enzymes including purine-nucleoside phosphorylase, adenine phosphoribosyltransferase, and adenosine nucleosidase caused significantly reduced levels of adenine as well. These findings suggest that PCN can lead to associated nitrogen metabolism disorders. **Figure 5I** shows that in the arachidonic acid pathway, the changes of glutathione peroxidase, arachidonate 5-lipoxygenase, cytochrome P450 family 2 subfamily J, long-chain fatty acid omega-monooxygenase, and hepoxilin A3 hydrolase can cause the upregulation of the metabolites 15(S)-HETE, 5-HPETE, 11,12,15-THETA, 20-HETE, and tnoxilin A3, respectively, indicating that PCN treatment leads to dysregulation of multiple substances in this pathway.

### Combined transcriptome and metabolome analysis

**Figure 6:**
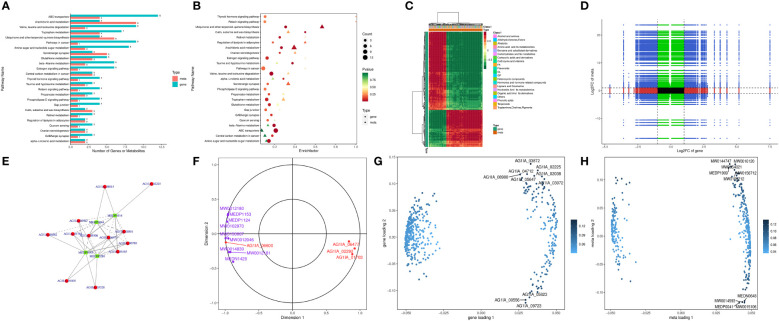
The antifungal molecular mechanisms of the antifungal compound PCN as revealed by the transcriptome and metabolome combined analysis. **(A)** bar graph of KEGG pathway enrichment analysis; **(B)** bubble chart of KEGG pathway enrichment analysis; **(C)** heatmap analysis; **(D)** nine-quadrant diagram of correlation analysis; **(E)** correlation network of differential genes and metabolites; **(F)** canonical correlation analysis (CCA); **(G)** gene loadings plot; **(H)** metabolite loadings plot.

Combined transcriptome and metabolome analysis revealed 25 KEGG pathways shared by the two, with the most prominent being ABC transporters, arachidonic acid metabolism, valine, leucine and isoleucine degradation, tryptophan metabolism, and ubiquinone and other terpenoid-quinone biosynthesis (**Figure 6A**, **B**), indicating possible impairment of the above pathways by PCN. The number of metabolites was higher than the number of genes in pathways such as arachidonic acid metabolism, as well as ubiquinone and other terpenoid-quinone biosynthesis, which was reversed in pathways such as valine, leucine and isoleucine degradation and tryptophan metabolism. This phenomenon suggested that within the same shared pathway, one gene may direct one metabolite or multiple metabolites, and one metabolite may also be directed by multiple genes.

**Figure 6C** shows that the genes and metabolites could be divided into two categories with one category having more genes than the other. In the first category, genes and most metabolites were negatively correlated, while in the second category, genes and metabolites were positively correlated, suggesting low correlation between most genes and metabolites. As shown in **Figure 6D**, through expression trend analysis, the quantitative values of genes and metabolites in all samples could be divided into nine quadrants. Among these, genes and metabolites in the fifth quadrant were all non-differentially expressed, without excavatable genes and metabolites. The differential expression of genes and metabolites in the third quadrant and seventh quadrant had similar consistent patterns, suggesting that these genes and metabolites were mostly positively correlated, and metabolite expression changes may be caused by positive gene regulation. In contrast, the differential expression of genes and metabolites in the first quadrant and ninth quadrant had opposite patterns, suggesting that these genes and metabolites were mostly negatively correlated, and metabolite expression changes may be negatively regulated by genes. Genes and metabolites with unchanged expression were found in the second, fourth, sixth, and eighth quadrants with no excavatable information. As is shown in **Figure 6E**, glucose-6-phosphate isomerase (AG1IA_02400) played the most crucial role in all gene and metabolite relationships, directing the expression of a variety of metabolites. Glucose-6-phosphate isomerase (AG1IA_02400) was positively correlated with the metabolite arbutin 6-phosphate (MW0145230), which may positively modulate the effect exerted by this gene on metabolite expression. In contrast, glucose-6-phosphate isomerase (AG1IA_02400) was negatively correlated with L-lactic Acid (MEDN0325) and arbutin (MW0015892), which may indicate the negative modulation of this gene during metabolite expression. In addition, aldehyde dehydrogenase (AG1IA_03762) and arbutin (MW0015892) were positively correlated, which may also indicate positive modulation of this gene during metabolite expression. Canonical correlation analysis (CCA) is a multivariate statistical method using the correlation between composite variable pairs to reflect the overall correlation between two groups of indicators. Results from CCA for the differential genes and differential metabolites in each pathway showed that aldehyde dehydrogenase (AG1IA_03762) and arbutin (MW0015892) clustered within the same region far from the origin while close to each other, indicating that the two were highly correlated. Glucose-6-phosphate isomerase (AG1IA_02400) and arbutin 6-phosphate (arbutin-6P, MW0145230) also gathered within the same area, which were far from the origin and had close distance to each other, implying a high correlation between the former and the latter as well (**Figure 6F**). Two-way Orthogonal PLS (O2PLS) is an unsupervised modeling method mainly used in integrated analysis of two datasets, which can objectively describe a system if a correlation trend exists between two datasets and avoids false positive correlations as much as possible at the source. We constructed a O2PLS model through selecting all differential genes and differential metabolites, preliminarily judged the variables with high correlation and weight in these different datasets, and identified the important variables impacting other omics datasets. **Figure 6G** shows that metabolomics was greatly impacted by upregulated DEGs including zinc-binding dehydrogenase domain-containing protein (AG1IA_03872), ABC transporter (AG1IA_02225), high mobility group (HMG) box domain-containing protein (AG1IA_04712), glutaredoxin domain-containing protein (AG1IA_02038), and salicylate 1-monooxygenase (AG1IA_08900), as well as by the downregulated DEG tryptophan 2,3-dioxygenase (AG1IA_00556), among which zinc-binding dehydrogenase domain-containing protein (AG1IA_03872) was the most influential. Conversely, our genomics dataset was largely affected by upregulated metabolites, including Ala-Thr-Ile-Lys (MW0144747), beta-glycyrrhetinic acid (MW0016120), ingenol (MW0054021), 1,2-cyclopentanedione (MEDP1909), Ser-Gln-Leu-Lys (MW0156712), and Phenylalanyl-Serine (MW0109212), as well as by downregulated metabolites, including 2,5-dihydroxybenzaldehyde (MEDN0648), 5(6)-epoxy-8Z,11Z,14Z-eicosatrienoic acid, methyl ester (MW0014593), Ala-Ala (MEDP0041), and 7(14)-bisabolene-2,3,10,11-tetrol (MW0015106), among which Ala-Ala (MEDP0041) was the most influential (**Figure 6H**).

As is shown in **Supplementary Table 5**, the differential gene leukotriene-A4 hydrolase in the arachidonic acid metabolism pathway was substantially upregulated, and its associated metabolites were also significantly upregulated with high correlation coefficients, where this gene probably played a positive regulatory role. Alpha/beta hydrolase family domain-containing protein and fatty acid oxygenase were markedly downregulated, whereas their corresponding metabolites were significantly upregulated with low correlation coefficients, and it was possible that the genes exerted negative modulatory effects. The differential genes fungal specific transcription factor domain-containing protein and NADH-quinone oxidoreductase in the pathway of ubiquinone and other terpenoid-quinone biosynthesis were also significantly upregulated, and their corresponding metabolite 6-Geranylgeranyl-2-methylbenzene-1,4-diol (MW0143834) was significantly upregulated as well, indicating these genes may positively modulate this pathway. In the valine, leucine and isoleucine degradation pathway, enoyl-CoA hydratase, dehydrogenase groES-like, and domain-containing protein were upregulated, with their corresponding metabolites significantly upregulated as well, meaning they may have been positively regulated by these genes. Adenosine deaminase in the purine metabolism pathway was significantly upregulated, and its five corresponding metabolites were upregulated as well. Conversely, downregulation of this compound had the corresponding metabolites downregulated as well, suggesting that this gene possessed dual function for positive and negative regulation. In the metabolic pathway of tryptophan metabolism, NADPH–cytochrome P450 reductase, aldehyde dehydrogenase, P450 family fatty acid hydroxylase, and cytochrome P450 domain-containing protein were downregulated, and their corresponding metabolites such as 2-picolinic acid and L-tryptophan were also downregulated, where these genes likely played positive regulatory roles.

In the amino sugar and nucleotide sugar metabolism pathway, glucose-6-phosphate isomerase and carbohydrate esterase family 4 proteins were significantly upregulated, with their metabolites being upregulated as well, suggesting a positive modulation of these genes. In contrast, glycosyltransferase family 2 protein was downregulated while its metabolites were significantly upregulated, implying a negative modulation of this gene. The above research results indicate that genes may exert only positive or negative regulation of a given pathway, but it was also possible that multiple regulations existed simultaneously.

### Effect of PCN on the biomass of *R. solani* AG1IA

**Figure 7:**
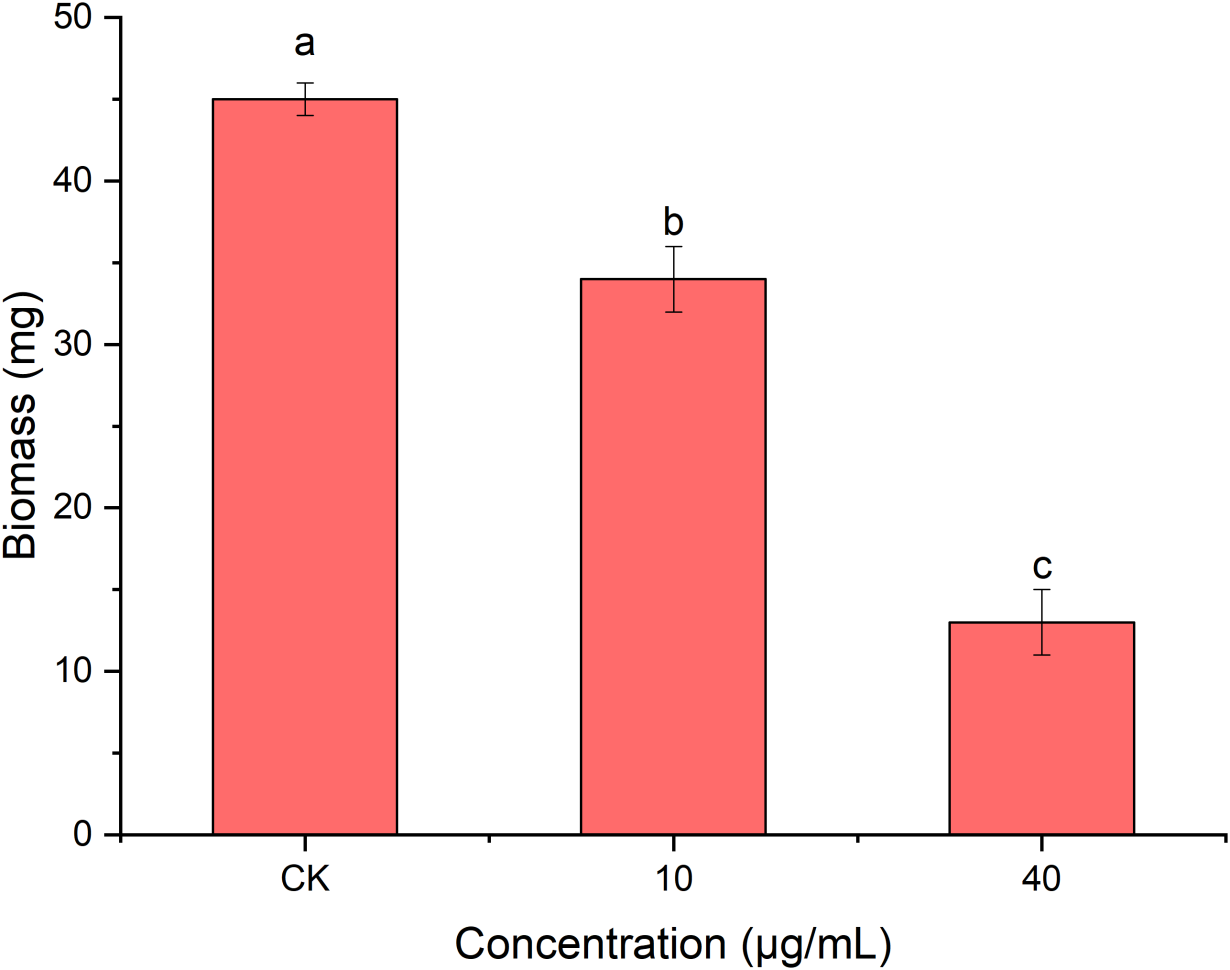
Effect of the antifungal compound PCN on the biomass of *R. solani* AG1IA. Different lowercase letters indicate significant differences (*P < 0.05*).

As shown in **Figure 7**, the mycelial biomass of *R. solani* AG1IA treated with PCN was decreased compared to controls. The higher the concentration of PCN, the less the mycelial biomass. When the concentration of PCN was 40 μg/mL, its biomass was only 0.29 times that of the control biomass. The results of variance analysis revealed that the mycelial biomass of *R. solani* AG1IA differed significantly between different treatments (*P < 0.05*), indicating a certain inhibitory effect of PCN on the amount of *R. solani* AG1IA mycelium.

### Effect of PCN on the protein content of *R. solani* AG1IA

**Figure 8:**
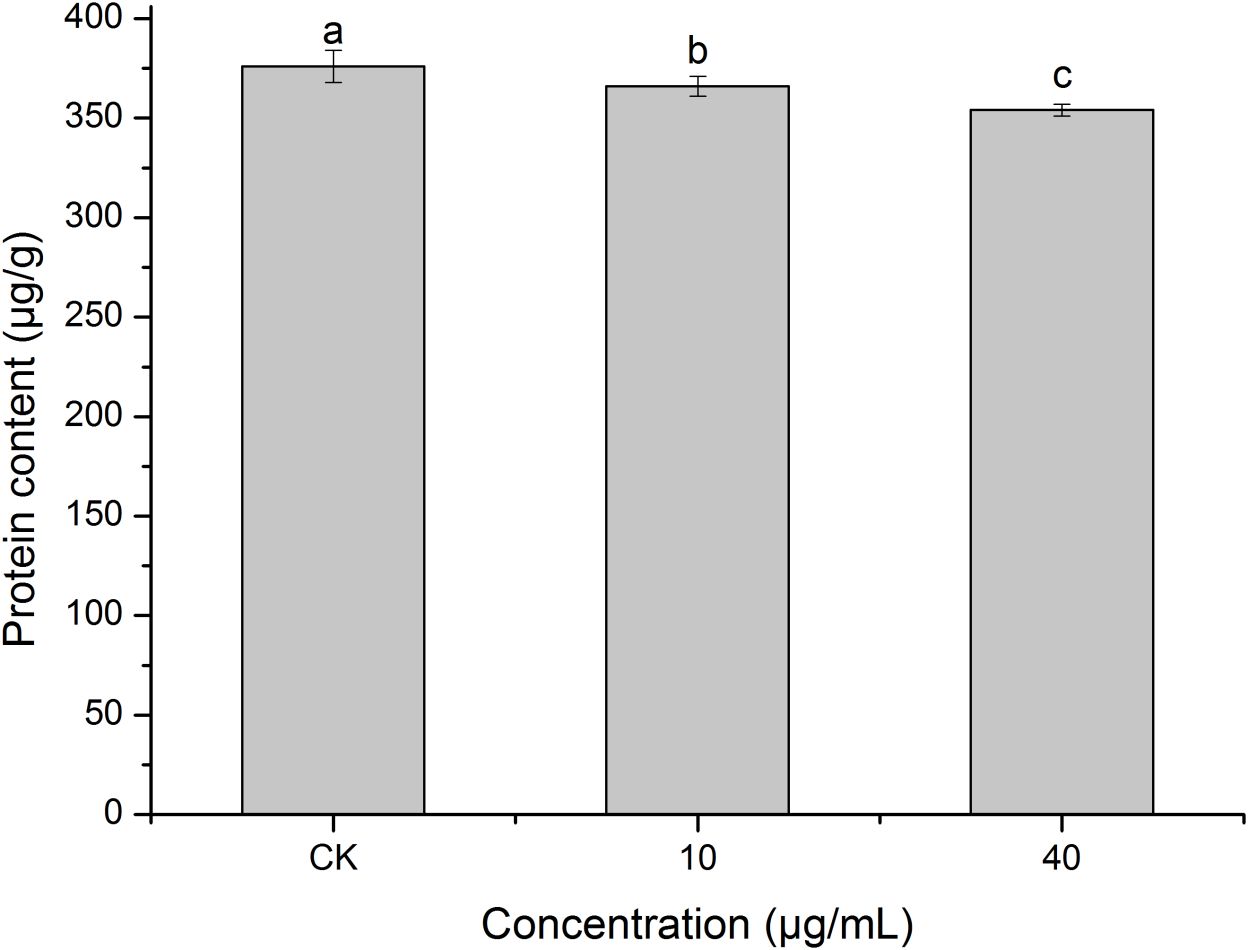
Effect of the antifungal compound PCN on the protein content of *R. solani* AG1IA. Different lowercase letters indicate significant differences (*P < 0.05*).

Protein is the basic substance for life activities (Chang, 2021). **Figure 8** shows that PCN decreased the protein content of *R. solani* AG1IA. The protein contents of the treatment groups were lower than that of the control group, and the differences were significant between distinct treatments (*P < 0.05*). The higher the concentration of PCN, the lower the protein content. The protein content was 354 μg/g at a PCN concentration of 40 μg/mL, which was 0.97 times that of the lowest concentration. These results indicate that PCN suppressed the protein production of *R. solani* AG1IA.

### Effect of PCN on the extracellular pH of *R. solani* AG1IA

**Figure 9:**
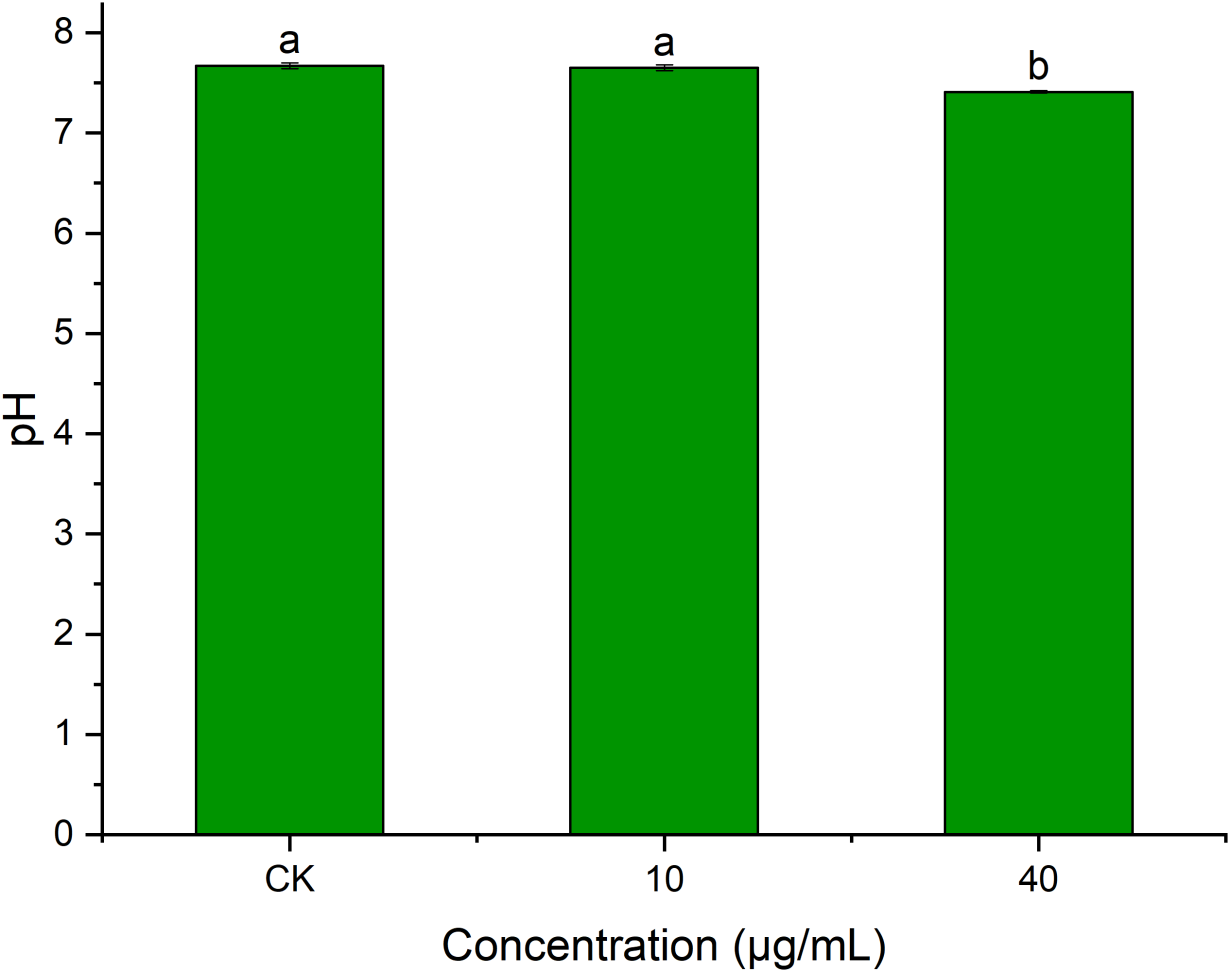
Effect of the antifungal compound PCN on the extracellular pH of *R. solani* AG1IA. Different lowercase letters indicate significant differences (*P<0.05*).

pH plays an important role in transmembrane transport across cell membranes, enzymatic activities, and biomolecular synthesis (Xue et al., 2015). **Figure 9** shows that PCN had an impact on *R. solani* AG1IA extracellular pH. The pH at a PCN concentration of 40 μg/mL was significantly lower than that of the control group (*P* < 0.05). The higher the concentration of PCN, the lower the observed pH. The pH of the highest PCN concentration was 0.95 times that of the lowest concentration. These indicated that PCN could decrease the extracellular pH of *R. solani* AG1IA.

### Effect of PCN on the enzymatic activities of *R. solani* AG1IA

**Figure 10:**
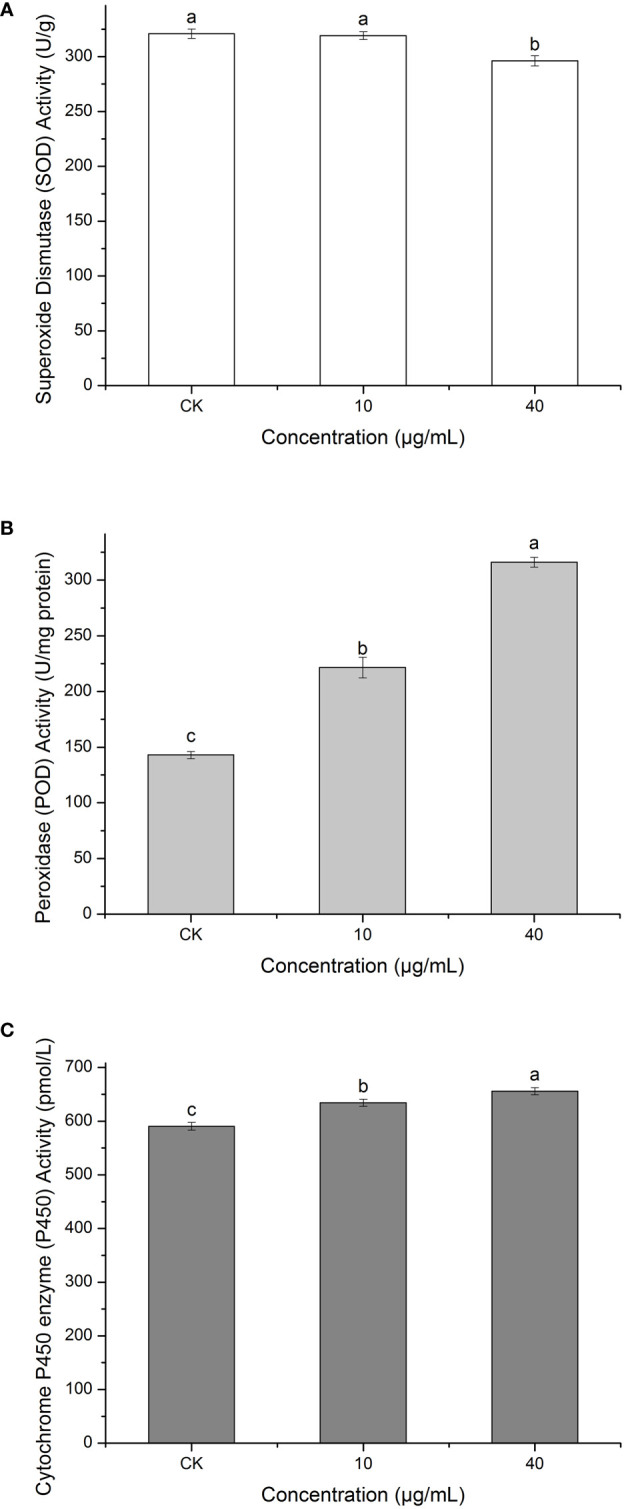
Effect of the antifungal compound PCN on the enzymatic activities of *R. solani* AG1IA. **(A)** superoxide dismutase (SOD); **(B)** peroxidase (POD); **(C)** cytochrome P450 monooxygenase; different lowercase letters indicate significant differences (*P < 0.05*).

Superoxide dismutase (SOD) can defend against oxygen toxicity, enhance an organism’s immunity, and prevent apoptosis (Chen and Liu, 1996). As is shown in **Figure 10A**, PCN could reduce the enzymatic activity of SOD in *R. solani* AG1IA. The treatment groups had significantly lower SOD enzymatic activities than the control group, which was significantly different (*P* < 0.05). The higher the concentration of PCN, the lower the SOD activity. The SOD activity at a PCN concentration of 40 μg/mL was 0.92 times that at 10 μg/mL. These suggested that PCN could weaken the SOD activity of *R. solani* AG1IA.

Peroxidase (POD) is an oxidoreductase related to stress, which is widely present in living organisms, is involved in various physiological and biochemical processes and plays critical roles in biological development and protection against biological disturbances (Wei et al., 2021). **Figure 10B** shows that the *R. solani* AG1IA in the treatment groups had higher POD enzymatic activity than the control group. The higher the concentration of PCN, the higher the POD activity observed. When the PCN concentration was 40 μg/mL, the POD activity was 2.21 - fold that in the control group. The results of variance analysis showed that the differences between distinct treatments were not significant (*P < 0.05*). The above experimental results suggested that PCN could increase the POD activity of *R. solani* AG1IA.

Cytochrome P450s (P450) are a type of monooxygenases possessing cysteine-heme structures. In the biological synthesis and modification procedures of multiple fungal-derived drug molecules, these enzymes participate in multi-step oxidation reactions (Meunier et al., 2004). **Figure 10C** shows that PCN had an impact on the enzymatic activity of cytochrome P450. The *R. solani* AG1IA in the treatment groups had higher P450 activities than the control group, which was significantly different (*P*<0.05). The higher the concentration of PCN, the higher the P450 enzymatic activity. When the PCN concentration was 40 μg/mL, the P450 activity was 1.03 - fold that at 10 μg/mL. This implied that PCN could improve the P450 enzymatic activity of *R. solani* AG1IA.

### Binding mechanisms of PCN to key receptors of *R. solani* AG1IA

In this study, we constructed protein models for NADPH nitrite reductase (AG1IA_09411), ATP-binding cassette transporter (AG1IA_00780), alpha/beta hydrolase family domain-containing protein (AG1IA_02292), and NADPH–cytochrome P450 reductase (AG1IA_03629). Their overall quality factor scores for ERRAT assessed by SAVES were 79.82, 91.01, 85.07, and 80.56, respectively. VERIFY3D evaluation results showed that NADPH nitrite reductase (AG1IA_09411) and NADPH–cytochrome P450 reductase (AG1IA_03629) had 86.34% and 81.33% of residues with averaged 3D-1D score ≥ 0.2, fully compliant with modeling requirements, while the ATP-binding cassette transporter (AG1IA_00780) and alpha/beta hydrolase family domain-containing protein (AG1IA_02292) just barely met the modeling requirements. Thus, the receptor structures constructed by this method were basically reliable and could be subjected to subsequent investigation.

**Figure 11:**
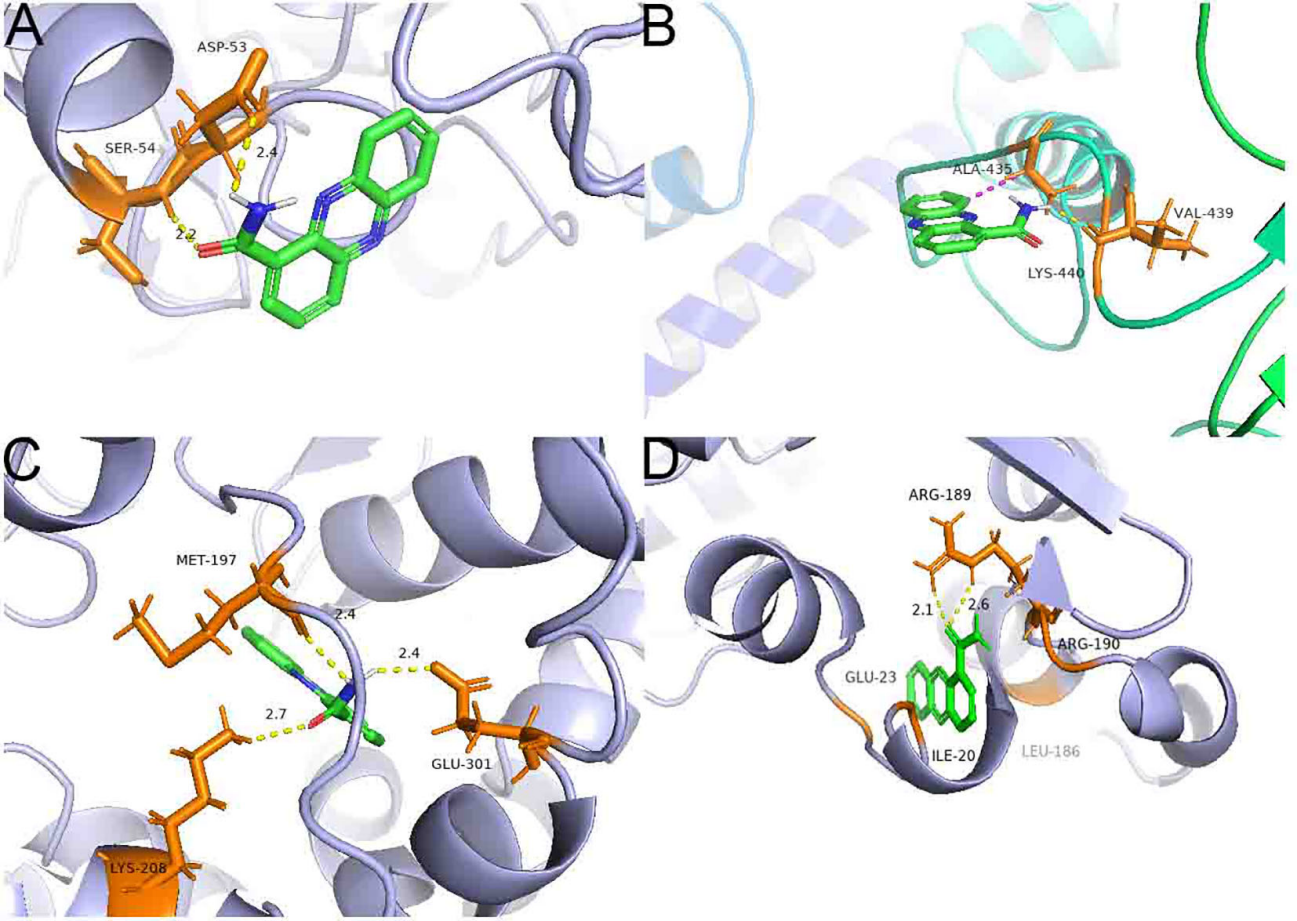
Binding complexes of PCN with key receptors in *R. solani* AG1IA. **(A)** NADPH nitrite reductase; **(B)** ATP-binding cassette transporter; **(C)** alpha/beta hydrolase family domain-containing protein; **(D)** NADPH–cytochrome P450 reductase.

Inverse molecular docking was employed to study the binding of PCN with NADPH nitrite reductase (AG1IA_09411), ATP-binding cassette transporter (AG1IA_00780), alpha/beta hydrolase family domain-containing protein (AG1IA_02292), and NADPH–cytochrome P450 reductase (AG1IA_03629) receptors. **Figure 11** shows that PCN could dock with NADPH nitrite reductase (AG1IA_09411), ATP-binding cassette transporter (AG1IA_00780), alpha/beta hydrolase family domain-containing protein (AG1IA_02292), and NADPH–cytochrome P450 reductase (AG1IA_03629). Among these, NADPH–cytochrome P450 reductase (AG1IA_03629) had the best binding effect, with a binding free energy of −5.93 kJ·mol^−1^. NADPH nitrite reductase (AG1IA_09411) was the second, with a binding free energy of −4.61 kJ·mol^−1^. Alpha/beta hydrolase family domain-containing protein (AG1IA_02292) was third with a binding free energy of −4.17 kJ·mol^−1^. ATP-binding cassette transporter (AG1IA_00780) had the worst binding effect, with a binding free energy of only −3.29 kJ·mol^−1^. PCN interacted with two amino acids labeled in orange in the NADPH nitrite reductase (AG1IA_09411) model, resulting in hydrogen bond interactions with SEP-54 and ASP-53, whose hydrogen bond distances were 2.2 and 2.4, respectively (**Figure 11A**). PCN interacted with three amino acids labeled in orange in the ATP-binding cassette transporter (AG1IA_00780) model, which had hydrophobic interactions with ALA-435, hydrogen bond interactions with VAL-439, and π bond interactions with LYS-440 (**Figure 11B**). PCN interacted with three amino acids labeled in orange in the alpha/beta hydrolase family domain-containing protein (AG1IA_02292) model, which formed hydrogen bond interactions with GLN-301, LYS-208, and MET-197, whose hydrogen bond distances were 2.4, 2.7, and 2.4, respectively (**Figure 11C**). PCN interacted with five amino acids labeled in orange in the NADPH–cytochrome P450 reductase (AG1IA_03629) model, which formed hydrophobic interactions with GLU-23, LIE-20, PHE-186, and ARG-190 as well as hydrogen bond interactions with ARG-189, and whose hydrogen bond distances were 2.1 and 2.6, respectively (**Figure 11D**).

### Model of molecular mechanisms PCN inhibits in *R. solani* AG1IA

**Figure 12:**
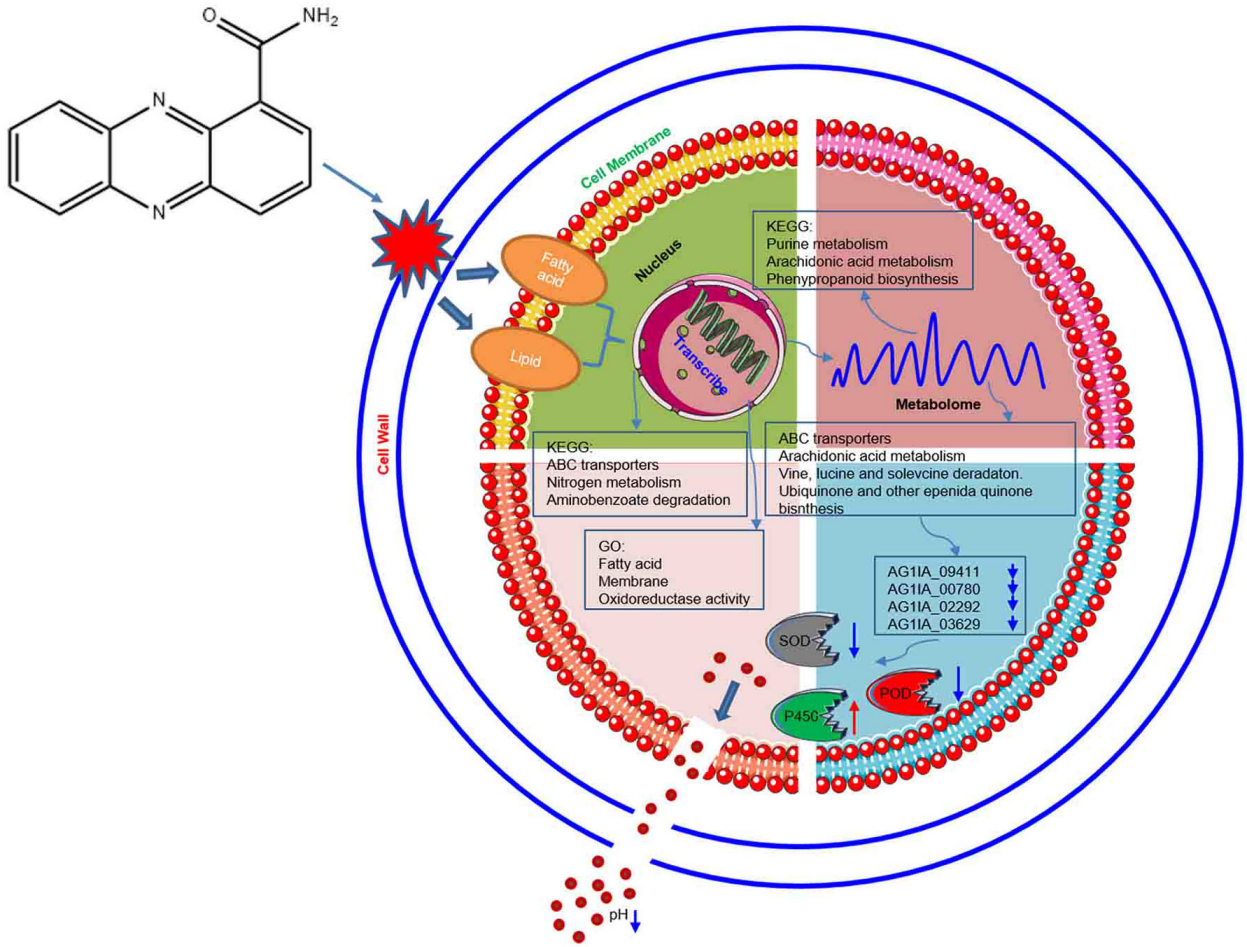
Model of molecular mechanisms *via* which the antifungal compound PCN inhibits *R. solani* AG1IA.

As shown in **Figure 12**, through damaging the cell wall of *R. solani* AG1IA, focusing on impairing the cell membrane, PCN thereby causes transcriptional abnormalities. In KEGG pathways, the enrichments for ABC transporters, nitrogen metabolism, and aminobenzoate degradation were primarily influenced. The GO functions mainly affected were fatty acid, membrane, and oxidoreductase activity. On the basis of these findings, metabolism was dysregulated. In KEGG pathways, purine metabolism, arachidonic acid metabolism, and phenypropanoid biosynthesis were the major metabolic pathways impaired. Results of combined transcriptome and metabolome analysis revealed that PCN mainly had impacts on the *R. solani* AG1IA pathways such as ABC transporters, arachidonic acid metabolism, valine, leucine and isoleucine degradation, and ubiquinone and other terpenoid-quinone biosynthesis. The downregulated genes of interest, including NADPH nitrite reductase (AG1IA_09411), ATP-binding cassette transporter (AG1IA_00780), ALPHA/BETA hydrolase family domain-containing protein (AG1IA_02292), and NADPH–cytochrome P450 reductase (AG1IA_03629), were selected for modeling, molecular docking, and investigation of the mode of action. It was found that PCN could interact well with the corresponding protein structures of all of the above genes, which may be potential binding sites. Subsequent assays confirmed that PCN decreased SOD enzymatic activity, while increasing the activities of POD and P450, and could also reduce extracellular pH.

## Discussion

The transcriptome is the bridge for studying the genetic information expressed by the genome and the biological functions of the proteome, which plays important roles in studies such as those uncovering new genes, discovering novel targets, and verifying gene functions (Xu et al., 2020). The present study explored for the first time from the transcriptome perspective, the molecular mechanisms by which PCN inhibits *R. solani* AG1IA. Our results indicate that PCN primarily had impact on several pathways, including ABC transporters, nitrogen metabolism, and aminobenzoate degradation, in the *R. solani* AG1IA strain. ABC transporters are one of the largest families of membrane transport proteins, which can bind ATP to utilize energy to drive the transport of various molecules, sequester toxic hydrophobic compounds into specific organelles, and also channel their extracellular secretion (Davidson and Chen, 2004; Chen et al., 2021). Damage to this pathway has been implicated in cell membrane metabolic disorders. Nitrogen is an essential element that makes up living organisms and can provide nutrients for the growth of microorganisms. The impact of drugs on nitrogen metabolism can indirectly cause microbial dysplasia (Chen, 2021). The results from this study confirmed that PCN affected the fungal nitrogen metabolism pathway, resulting in metabolic dysregulation, nutritional imbalance, and growth abnormality of this pathogen. Secondly, PCN considerably affected several functions, including lipids, fatty acid oxidation, and cell membrane components, and also significantly reduced the expression of key genes in cell wall, cell membrane, and energy metabolism processes, such as glycosyltransferase family 2 protein (AG1IA_09260 and AG1IA_07549), fatty acid oxygenase (AG1IA_01703), acetyl-CoA acetyl transferase (AG1IA_01871), ABC transporter domain-containing protein (AG1IA_03597), and P5-ATPase domain-containing protein (AG1IA_08254), implying that these might be potential action targets of PCN. These study results differ from those in Pute et al., which may be attributable to distinct research approaches respective to each other (Tupe et al., 2015). In addition, the results of this study revealed that PCN could destroy the cell wall, which was consistent with the research perspectives in Xiang et al., demonstrating that the cell wall could possibly be a PCN target (Xiang et al., 2018). However, our study also found that there were also a portion of genes exhibiting upregulated expression when PCN acted on the same type of function or the same enzyme. For instance, the upregulated phenylacetyl-CoA ligase (AG1IA_09040) expression may accelerate the catalysis of phenylacetic acid, or the upregulated enoyl-CoA hydratase (AG1IA_02110) expression may promote the degradation of lipids and amino acid substances. Additionally, the upregulated ABC transporter (AG1IA_06165) expression may raise the substance amount of transmembrane transport. These results suggest that to resist the damage PCN inflicts, this pathogen turned on its self-protection mechanisms and enhanced gene expression, which were all related to resistance (Yu et al., 2011; Beis, 2015). Moreover, this study also confirmed that PCN was likely a multi-target and multi-process fungicidal compound, which was similar to the phenazine germicidal compounds previously reported, indicating that this pathogen does not easily develop resistance during PCN usage (Yin et al., 2021). Additionally, the *R. solani* AG1IA stain can secret cell wall-degrading enzymes and toxins to destroy the cell walls of hosts, thereby killing host cells, obtaining nutrients needed for its own growth and development, and assisting its expansion within host (Chen, 2020). Our study results verified that PCN downregulated the expression of gene associated with *R. solani* pathogenicity such as glycoside hydrolase family 61 protein (AG1IA_06330), manifesting in a loss of pathogenicity in *R. solani* by PCN. However, some topics remain unclear and need to be explored in the future. Were these disease-causing genes the key genes, or which pathogenic genes played a leading role and others a supporting role? Does PCN repress these downregulated causative genes simultaneously or in a sequential manner? What downstream physiological and biochemical processes would result from pathogenic gene downregulation? Overall, the present research findings have provided a new insight for the explanation of antifungal molecular mechanisms of PCN, enriching the theory behind the antimicrobial mechanisms of phenazine compounds, and also has laid the foundation for later pharmaceutical design, novel target discovery, and in-depth and systematic studies of compounds of this type. Of course, this study was not able to analyze all of the data from transcriptome testing, and there is still much work to be followed up on. For example, some gene functions remain obscure, and the genes with the most upregulated or downregulated expression were not necessarily target genes, which requires further validation through gene knockout or RNA interference (RNAi) techniques. However, since RNAi has only been conducted in a few microorganisms such as *Botrytis cinerea* or *Aspergillus* and large-scale research is in infancy in these organisms, there may be some difficulty taking advantage of this technique for gene function verification. There are also some genes with false positive issues that need one by one examination, which will require many years to complete (Nakayashiki and Nguyen, 2008; Li et al., 2010; Chang et al., 2012).

Transcriptomic sequencing can yield a large quantity of DEGs and regulatory networks, making it challenging to ascertain the crucial pathways in a dataset within a short time and the structure controlling crucial pathways cannot be identified as well. Metabolites, however, are the ultimate manifestation of life activities, and minor changes in phenotypic traits are exponentially magnified at metabolic levels, which allows utilizing the metabolome to reflect altered phenotypes. Metabolomics is a new method to study metabolic product changes within pathogens, and can be used to explore possible action mechanisms through analyzing associated metabolic pathways for the dynamics of a particular molecule or metabolite within pathogens. We found in this study that the number of upregulated differential metabolites was higher than the number of downregulated ones in PCN-treated fungus, which may be because to resist the stress induced by PCN, the pathogen accelerates the production of many metabolites, *via* the forms of either metabolite dilution or PCN toxicity alteration, to reduce the inhibitory effect of PCN on this pathogen. Secondly, we also found that PCN had the greatest impact on the fungal purine metabolic pathway. This metabolic pathway is closely related to nucleic acid synthesis and decomposition, as well as intracellular second messenger synthesis. It has been found to be associated with many types of inflammation or diseases in animals, while there is no research yet regarding the abnormality of this metabolic pathway and fungal diseases, meaning the exact relationship of remains to be further investigated. Moreover, PCN also greatly affected the fungal arachidonic acid metabolic pathway. These substances exist chiefly in the form of phospholipids on the fungal cell membrane, which can function as second messengers in cells or can also promote or amplify other second messenger systems, such as cAMP and cGMP. Thus, cell signal transduction and other related pathways play important roles during the suppression of *R. solani* AG1IA by PCN. To sum up, in these pathways, the differential metabolites also mostly exhibited upregulated trends, implying that the metabolites of this pathogen were mostly associated with resistance. However, most of the umbelliferone and coumarin scopolin compounds in the phenylpropanoid biosynthesis metabolic pathway possess antifungal effects, whose upregulation denotes accelerated apoptosis of pathogens (Kai et al., 2006; Pan et al., 2017). This was consistent with the results that the more resistant a plant is to a pathogen, the more active the phenylpropanoid metabolism. Nevertheless, there is limited literature regarding the impact of phenylpropanoid pathway on fungi, and thus it remains to be further studied how the phenylpropanoid biosynthesis pathway strengthens the effect of PCN in *R. solani* AG1IA. Tyrosine is a very important substance in the metabolic pathways of thiamine metabolism and melanogenesis, which is a substance that is capable of forming melanin. Melanin can protect fungi from the harms of environmental stress, enhance nutrition, and promote the development of reproductive structures such as mycelium. Through producing melanin, many fungi block harmful radiation of various wavelengths, scavenge reactive oxygen species, sequester metal ions, and strengthen their cell wall (Wang et al., 2010). Tyrosine content was significantly downregulated in this study, impacting three pathways including phenylpropanoid biosynthesis, thiamine metabolism, and melanogenesis. Among them, melanogen is a precursor to generate melanin. It was thus speculated that PCN affects melanin production in *R. solani* AG1IA through inhibiting tyrosine synthesis, thereby compromising the capacity of *R. solani* AG1IA to cope with various environmental stresses, ultimately influencing its normal growth. Additionally, O-β-D-glucosyl-*trans*-zeatin plays a vastly prominent role in the metabolic pathway of zeatin biosynthesis, which is a cytokinin produced by many fungi. Research has shown that pathogenic fungus-derived cytokinin was the key effector for dampening host defenses (Chanclud et al., 2016). In this study, downregulated O-β-D-glucosyl-*trans*-zeatin signified possibly reduced resistance of *R. solani* AG1IA against host defense, thereby decreasing its infection of plants. Moreover, we also found in this study that tryptamines, cholines, and pigments were the most critical metabolites among the differential metabolites of PCN-treated mycelium, which were positively correlated with multiple substances such as amino acids, nucleic acids, and organic acids, while negatively correlated with a small number of metabolites. These results reveal that these types of metabolites play regulatory roles during fungal growth and development, and whose deficiency probably impact particular life activities of this pathogen. Furthermore, results of this study showed that the most differentially upregulated or downregulated metabolic products induced by PCN in this pathogen were not necessarily its acting targets. Since there were still plenty of physiological processes from the targeted genes to metabolites, which are very complicated and beyond full clarification, follow-up verifications are absolutely required. Due to the limitations of the experimental conditions, there are many questions remaining to be explored. For instance, the important metabolites discovered in this study were not necessarily the most principal ones. Conversely, those metabolites not remarkably downregulated may also play important roles or be truly non-functional. Whether or not the exact function was present and what effects were exerted by PCN treatment remain to be examined.

Are the genetic changes at the level of transcripts genuinely the key genes determining the phenotypic changes resulting from PCN treatment? This question demands answering through combined analyses of the transcriptome and the metabolome. Transcriptome and metabolome integrated analysis can explore biological questions and potential regulatory network mechanisms fulfilling the two layers including “cause” and “effect” at the same time and have mutual validations. These studies screen and identify critical genes, metabolites, and metabolic pathways from massive dataset, analyzing in depth the macroscopic developmental process of biological systems, explaining the complexity and integrity of biological processes, and can provide more evidence on the antifungal molecular mechanisms of PCN. Integrated analysis revealed that PCN had the greatest impact on pathways including ABC transporters, arachidonic acid metabolism, valine leucine and isoleucine degradation, and ubiquinone and other terpenoid-quinone biosynthesis in this pathogen. However, among these strongly associated pathways, the number of differential genes and the number of differential metabolites were not augmented simultaneously, with the number of differential genes being higher than the number of differential metabolites in some metabolic pathways, while the number of differential genes being lower than the number of differential metabolites in some other metabolic pathways, which may have been related to complicated life activities. From the transcriptional level to the metabolic level, multiple processes such as translation, modification, and biochemical reactions are often accompanied, and alterations in any procedure can possibly cause vast changes in subsequent metabolites. Secondly, this study found weak correlations between the most significantly enriched pathways from the transcriptome and the ones from the metabolome in PCN-treated pathogen, meaning a certain gap existed between connecting the two. This may be related to the complexity of physiological and biochemical activities, and an analysis of parameters may also be required. However, due to limited time and efforts, the present study only focused on data with significant differences, meaning a lot of data has not yet been mined, such as data not reaching significance in combined analysis or data without strong association between the two. These unexcavated data may also be important and will be useful for emphasized exploitation in the future. Moreover, this study also implied that despite downregulated expression of genes related to cell wall, cell membrane, energy metabolism, and pathogenicity in PCN-treated pathogen, their associated metabolites were not found in the metabolome. For example, the substances such as glucan, chitin, ergosterol, inositol phosphate, and adenosine diphosphate (ADP), were all unchanged. This might be related to the fact that the blocked gene expression at the transcriptional level resulted in directly reduced metabolites at the metabolic level and insufficient enrichment amount (Yang and Yang, 1996; Zhu et al., 2007). In addition, due to the influence of technologies, it remains to be elucidated how PCN actually regulates upstream gene expression and causes changes in downstream metabolites. These antifungal mechanisms of PCN can only be revealed more systematically and comprehensively by employing mutual corroboration of genomics techniques, proteomics techniques, modifiedomics techniques, and multiple physiological and biochemical processes, with the goal of providing a more theoretical basis and practical foundation for the future application of this compound.

## Conclusion

The mechanisms by which PCN inhibits *R. solani* AG1IA may be through destroying the cell wall and damaging the cell membrane, thereafter, causing transcriptional abnormalities. Further study found that PCN could also cause metabolic disorders in this fungus, and PCN primarily impaired purine metabolism, arachidonic acid metabolism, and phenylpropanoid biosynthesis metabolic pathways. The results from combined transcriptome and metabolome analyses revealed that PCN mainly affected specific pathways in *R. solani* AG1IA, including ABC transporters, arachidonic acid metabolism, and valine, leucine and isoleucine degradation. Biomass test data implied that PCN decreased the mycelial biomass and protein content of *R. solani*. Results of enzymatic activity assays also indicated that PCN could reduce SOD activity, while increasing the activities of POD and cytochrome P450. Molecular simulation results revealed that PCN could interact well with several target protein structures, including NADPH nitrite reductase (AG1IA_09411), ATP-binding cassette transporter (AG1IA_00780), alpha/beta hydrolase family domain-containing protein (AG1IA_02292), and NADPH–cytochrome P450 reductase (AG1IA_03629), which may be potential binding sites of this agent. Furthermore, it was confirmed that PCN may affect the antioxidant system of this pathogen and decrease extracellular pH. These findings provide a broader perspective for the investigation of the molecular mechanisms by which PCN inhibits *R. solani* AG1IA.

## Data availability statement

The data presented in the study are deposited in the NCBI repository, accession number PRJNA892065. Further inquiries can be directed to the corresponding authors.

## Author contributions

YZ analyzed the data and wrote the manuscript. QL were responsible for study initiation and performed the experiments. CW contributed to the literature search, collection and assembly of data. SL supervised the project and grammar correction. All authors contributed to the article and approved the submitted version.

## Funding

This research was financially supported by the National Natural Science Foundation of China (32072473), the double first-class construction project of Hunan Agricultural University (SYL2019033).

## Conflict of interest

The authors declare that the research was conducted in the absence of any commercial or financial relationships that could be construed as a potential conflict of interest.

## Publisher’s note

All claims expressed in this article are solely those of the authors and do not necessarily represent those of their affiliated organizations, or those of the publisher, the editors and the reviewers. Any product that may be evaluated in this article, or claim that may be made by its manufacturer, is not guaranteed or endorsed by the publisher.
