# Supplementary material for: Transcriptomic and metabolomic analyses reveal the antifungal mechanism of the compound phenazine-1-carboxamide on Rhizoctonia solani AG1IA
Source: Front Plant Sci. 2022 Nov 22;13:1041733. doi: 10.3389/fpls.2022.1041733 (PMC9722969; doi:10.3389/fpls.2022.1041733)
Supplement: Supplementary file 1 [file DataSheet_1.pdf]

Supplementary Table 1 Oligonucleotide primers used in this study.

Gene Number	Primer name	Primer sequence
AG1IA_03629	F	CACCGTATCTTGATGCCTGC
	R	AGGCAAGTCGAGTGAAGTCA
AG1IA_09413	F	GAGCCTGTCGTGTTTTCCAG
	R	TTCGCCAACTCTAGTCAGCA
AG1IA_01412	F	TTCGTTCCCCTTGTCTCACA
	R	TATACCACCTGCAGCAACGA
AG1IA_03634	F	AGTTGTAGGGGACGGAAAGT
	R	CCTCTCACGTGGGCTATGAA
AG1IA_10228	F	CAAGAAGGACCTGCTGAACG
	R	CTTCATCACATGCGCCATCA
AG1IA_00780	F	GCTCTTTGAGGCGAGTCATG
	R	CATGTAAAGCCGCAACCAGA
AG1IA_06975	F	CTGACAAGTCCCTAGTGGCA
	R	GTGCTGTGCGAATGGTAGAG
AG1IA_01805	F	ATTCTCTGCACCATCCACCA
	R	ATTCAGCAGGGTTCTCGTCA
AG1IA_00881	F	TGCCGGGTTTGCTATCATTG
	R	TCCCGTGGATTGCTTACTGT
AG1IA_01784	F	GGGTATTCTAGTGTGGCCGA
	R	CTGTGAGTAGGGTTGGAGCA
