# Supplementary material for: Transcriptomic and metabolomic analyses reveal the antifungal mechanism of the compound phenazine-1-carboxamide on Rhizoctonia solani AG1IA
Source: Front Plant Sci. 2022 Nov 22;13:1041733. doi: 10.3389/fpls.2022.1041733 (PMC9722969; doi:10.3389/fpls.2022.1041733)
Supplement: Supplementary file 2 [file DataSheet_2.pdf]

Supplementary Table 2 Data quality statistics

Sample	Total_reads	Total_bases (G)	Q20(%)	Q30(%)	GC_content (%)
CK1	26445898	7.87	98.63	94.93	52.96
CK2	28483697	8.47	98.53	94.64	52.9
CK3	20342574	6.03	98.11	93.32	52.75
TM1	33161030	9.86	98.55	94.69	52.85
TM2	20040436	5.96	98.53	94.65	52.82
TM3	22001342	6.55	98.6	94.85	52.72

Note: CK: Control; TM:Treatment;
