# Supplementary material for: Transcriptomic and metabolomic analyses reveal the antifungal mechanism of the compound phenazine-1-carboxamide on Rhizoctonia solani AG1IA
Source: Front Plant Sci. 2022 Nov 22;13:1041733. doi: 10.3389/fpls.2022.1041733 (PMC9722969; doi:10.3389/fpls.2022.1041733)
Supplement: Supplementary file 3 [file DataSheet_3.pdf]

Supplementary Table 3 Differentially expressed genes related to the potential pathways of *Rhizoctonia solani*

Serial No		Gene Id	Log2 Fold Change	P value	Regulated
1	glycosyltransferase family 2 protein	AG1IA_09260	-1.35	3.11E-08	down
2		AG1IA_07549	-1.22	1.49E-07	down
3	chitin synthase 6	AG1IA_07339	-1.34	5.97E-10	down
4	chitin synthase G	AG1IA_04366	-1.23	4.57E-07	down
5	glycoside hydrolase family 16 protein	AG1IA_09291	-1.53	5.15E-08	down
6	alpha-1,3 glucan synthase	AG1IA_00910	-1.02	2.47E-05	down
7	fatty acid metabolic process	AG1IA_00780	-1.29	6.36E-08	down
8		AG1IA_09459	-1.21	0.000104038	down
9		AG1IA_01871	-1.06	0.000516877	down
10		AG1IA_06477	-1.02	1.02E-05	down
11		AG1IA_09252	-1.35	7.25E-08	down
12		AG1IA_01703	-1.53	5.36E-09	down
13		AG1IA_05287	-1.65	5.51E-08	down
14		AG1IA_02292	-1.92	6.29E-13	down
15		AG1IA_05255	-1.14	0.000200135	down
16		AG1IA_08166	-1.01	4.24E-05	down
17	arachidonic acid metabolic pathway	AG1IA_02292	-1.92	6.29E-13	down
18		AG1IA_06477	-1.02	1.02E-05	down
19		AG1IA_01703	-1.53	5.36E-09	down
20	fatty acid metabolic process	AG1IA_09040	1.22	0.000205491	up
21		AG1IA_01990	1.04	3.08E-05	up
22		AG1IA_02110	1.04	5.21E-05	up
23		AG1IA_04388	-0.15	0.583074306	down
24		AG1IA_05814	1.46	0.00229877	up
25	fatty acid oxidation	AG1IA_04388	2.19	3.45E-06	up
26		AG1IA_02110	1.04	5.21E-05	up
27	plasma membrane function	AG1IA_01398	1.07	0.004975028	up
28		AG1IA_03838	1.1	3.75E-05	up
29		AG1IA_04180	1.01	0.000722082	up
30		AG1IA_05884	1.15	1.65E-05	up
31	nitrogen metabolism	AG1IA_09410	-1.05	0.000526731	down
32		AG1IA_09412	-1.02	0.000141063	down
33		AG1IA_06435	-1.25	4.54E-08	down
34		AG1IA_09411	-1.3	0.00019976	down
35		AG1IA_09413	-1.36	0.000155179	down
36		AG1IA_01412	-1.77	2.19E-09	down
37	aminobenzoate degradation	AG1IA_01905	1.43	2.69E-07	up
38		AG1IA_04867	1.52	5.54E-09	up
39		AG1IA_04388	2.19	3.45E-06	up
40		AG1IA_09272	7.51	2.82E-136	up
41		AG1IA_04881	1.58	1.33E-11	up
42		AG1IA_00318	1.37	3.97E-07	up

43		AG1IA_03872	1.04	0.003954088	up
44		AG1IA_04388	2.19	3.45E-06	up
45	valine, leucine and isoleucine degradation	AG1IA_02110	1.04	5.21E-05	up
46		AG1IA_05942	1.07	2.58E-07	up
47		AG1IA_08405	1.69	0.00146793	up
48		AG1IA_01275	1.05	0.001738156	up
49	vacuole-mitochondrion	AG1IA_08254	-1.36	2.07E-09	down
50		AG1IA_03168	-1.05	7.43E-07	down
51		AG1IA_02536	-1.32	3.19E-06	down
52		AG1IA_08254	-1.36	2.07E-09	down
53		AG1IA_05862	-1.44	2.28E-07	down
54		AG1IA_04742	-1.01	0.000169418	down
55		AG1IA_06082	-1.02	0.000242978	down
56	ATPase activity	AG1IA_00780	-1.29	6.36E-08	down
57		AG1IA_06975	-1.74	1.77E-07	down
58		AG1IA_03597	-1.31	3.20E-06	down
59		AG1IA_03328	-1.74	3.42E-07	down
60		AG1IA_01805	-1.3	2.06E-05	down
61		AG1IA_01784	-1.55	1.15E-06	down
62		AG1IA_08015	1.59	6.34E-06	up
63	ABC	AG1IA_06165	1.72	1.39E-11	up
64		AG1IA_09306	2.3	1.24E-09	up
65		AG1IA_02225	1.48	3.55E-05	up
66		AG1IA_03192	-1.22	6.08E-06	down
67	pH	AG1IA_03187	1.29	1.63E-05	up
68		AG1IA_03188	1.03	0.002312429	up
69		AG1IA_00780	-1.29	6.36E-08	down
70		AG1IA_04529	-1.45	2.14E-08	down
71		AG1IA_05287	-1.65	5.51E-08	down
72		AG1IA_02292	-1.92	6.29E-13	down
73		AG1IA_09459	-1.21	0.000104038	down
74		AG1IA_05255	-0.18	0.476519579	down
75		AG1IA_08166	-1.01	4.24E-05	down
76		AG1IA_03762	-1.11	0.006180729	down
77		AG1IA_03903	-1.49	1.70E-05	down
78		AG1IA_08835	-1.04	0.004149793	down
79		AG1IA_03629	-1.7	1.05E-05	down
80		AG1IA_01412	-1.77	2.19E-09	down
81		AG1IA_09085	-2.65	3.34E-20	down
82		AG1IA_03634	-1.68	3.55E-10	down
83		AG1IA_08511	-1.29	2.95E-05	down
84		AG1IA_07677	-1.96	0.000177331	down
85		AG1IA_07059	-1.21	0.001196972	down
86	antioxidative	AG1IA_03903	-1.49	1.70E-05	down
87		AG1IA_09807	-1.08	0.000111992	down
88		AG1IA_08914	-1.06	0.005432118	down
89		AG1IA_06435	-1.25	4.54E-08	down
90		AG1IA_03629	-1.7	1.05E-05	down
91		AG1IA_08606	-1.15	0.000164274	down
92		AG1IA_01412	-1.77	2.19E-09	down

93		AG1IA_09085	-2.65	3.34E-20	down
94		AG1IA_10228	-1.76	7.72E-13	down
95		AG1IA_09040	1.22	0.000205491	up
96		AG1IA_08976	1.13	0.007972697	up
97		AG1IA_01788	3.74	1.78E-09	up
98		AG1IA_00127	1.14	6.47E-06	up
100		AG1IA_08900	1.32	0.005816182	up
101		AG1IA_09626	1.17	0.004562415	up
102		AG1IA_02038	2.68	1.06E-08	up
103		AG1IA_08597	2.34	1.88E-05	up
104		AG1IA_03269	1.61	0.000193748	up
105		AG1IA_06330	-2.06	2.10E-09	down
106		AG1IA_09291	-1.53	5.15E-08	down
107	pathogenicity	AG1IA_01834	-1.29	9.68E-06	down
108		AG1IA_00256	1.18	2.45E-07	up
109		AG1IA_02785	1.4	0.001280222	up
110		AG1IA_02474	4.46	5.71E-11	up
