# Supplementary material for: Transcriptomic and metabolomic analyses reveal the antifungal mechanism of the compound phenazine-1-carboxamide on Rhizoctonia solani AG1IA
Source: Front Plant Sci. 2022 Nov 22;13:1041733. doi: 10.3389/fpls.2022.1041733 (PMC9722969; doi:10.3389/fpls.2022.1041733)
Supplement: Supplementary file 4 [file DataSheet_4.pdf]

Supplement Table 4 Significantly different metabolites putatively identified by UPLC-MS/MS

Number	Index	Formula	Compounds	Log2FC	Type
1	MADN0586	C7H10O7	2-methyl citric acid	-2.19	down
2	MEDL01872	C19H30O3	[8]-Paradol	-1.34	down
3	MEDL01886	C6H10O3	3-Methyl-2-Oxovalerate	1.89	up
4	MEDL01915	C8H14O4	2-Propylglutaric acid	-2.07	down
5	MEDL02489	C15H12O5	Naringenin chalcone	16.21	up
6	MEDL02717	C12H20O3	12-Oxo-10E-Dodecenoic Acid	1.86	up
7	MEDN0051	C5H11NO3S	Methionine Sulfoxide	2.54	up
8	MEDN0056	C8H15NO3	N-Acetyl-L-Leucine	-2.61	down
9	MEDN0098	C6H5NO2	2-Picolinic Acid	-1.86	down
10	MEDN0140	C5H4N4O2	Xanthine	-1.07	down
11	MEDN0153	C10H14N5O7P	Adenosine 5'-Monophosphate	2.68	up
12	MEDN0165	C10H13N4O8P	Inosine 5'-monophosphate	2.42	up
13	MEDN0195	C8H10NO6P	Pyridoxal 5'-Phosphate	1.00	up
14	MEDN0280	C2H7NO3S	2-Aminoethanesulfonic Acid	-1.01	down
15	MEDN0293	C3H6O3	3-Hydroxypropanoic Acid	-1.14	down
16	MEDN0301	C9H8O4	Caffeic Acid	1.62	up
17	MEDN0320	C9H10O2	Hydrocinnamic Acid	-2.56	down
18	MEDN0325	C3H6O3	L-Lactic Acid	-1.14	down
19	MEDN0376	C18H34O4	9,10-DiHOME	1.58	up
20	MEDN0380	C16H30O2	FFA(16:1)	1.06	up
21	MEDN0385	C12H24O2	FFA(12:0)	1.15	up
22	MEDN0445	C10H12N2O8	Orotidine	-1.09	down
23	MEDN0480	C3H7O6P	Dl-Glyceraldehyde3-Phosphate	1.62	up
24	MEDN0485	C6H11Na3O12P2	D-Fructose-1,6-Biphosphate-Trisodium Salt	1.68	up
25	MEDN0586	C5H9NO3	N-Acetyl-L-alanine	-1.64	down
26	MEDN0602	C10H14N5O7P	deoxyguanosine 5'-monophosphate(dGMP)	2.68	up
27	MEDN0624	C10H14N5O10PS	5'-Adenylyl sulfate(APS)	2.45	up
28	MEDN0648	C7H6O3	2,5-Dihydroxybenzaldehyde	2.09	up
29	MEDN0658	C16H30O4	Hexadecanedioic acid	1.17	up
30	MEDN0733	C12H16O6	Phenyl-beta-D-glucopyranoside	12.21	up
31	MEDN0818	C5H9NO3	N-acetyl-beta-alanine	-1.60	down
32	MEDN1041	C18H30O4	9(S)-HpOTrE	1.02	up
33	MEDN1081	C18H34O4	12,13-DiHOME	1.58	up
34	MEDN1093	C7H12O5	2-isopropylmalate	1.15	up
35	MEDN1117	C17H27N3O17P2	Uridine-5'-diphospho-N-acetylgalactosamine disodium salt	1.17	up
36	MEDN1135	C19H40O3	Heparin	1.15	up
37	MEDN1179	C16H26O7	8-Epidermal glycoside	1.97	up
38	MEDN1226	C5H9NO3	2-amino-4-oxovaleric acid	-1.60	down
39	MEDN1236	C15H21N5O13P2	Cyclic ADP ribose	1.65	up
40	MEDN1241	C9H17O12P	2-(α-D-mannosyl) -3-phosphate glyceride	2.62	up
41	MEDN1255	C27H49O12P	LPI(18:2/0:0)	-1.07	down
42	MEDN1302	C6H14O12P2	D-Inositol-1,4-diphosphate	1.70	up
43	MEDN1324	C11H14O2	5-Phenylvaleric Acid	-11.50	down
44	MEDN1395	C9H10O2	(R)-(-)-2-Phenylpropionic Acid	-2.56	down
45	MEDN1429	C20H30O4	Prostaglandin B2	1.12	up
46	MEDN1475	C6H15O9P	Sorbitol 6-phosphate	-1.31	down
47	MEDN1501	C10H14N2O6	2'-O-methyluridine	14.49	up
48	MEDN1553	C10H14N4O11P2	Inosine diphosphate	2.08	up
49	MEDN1554	C2H6O4S	Isethionic acid	-1.07	down
50	MEDN1561	C17H34O4	MG(14:0/0:0/0:0)	-1.23	down
51	MEDN1586	C10H15N2O8P	Thymidine-5'-phosphate(dTMP)	2.30	up
52	MEDN1589	C12H20O4	Traumatic acid	3.05	up
53	MEDN1616	C16H29N3O4	Val-Pro-Leu	11.25	up
54	MEDN1654	C15H22O3	Gemfibrozil	-1.12	down
55	MEDN1669	C13H14N2O3	N-AcetyTryptophan	-1.76	down
56	MEDN1691	C9H14N4O4	His-Ser	2.09	up
57	MEDP0009	C9H11NO3	L-Tyrosine	-2.73	down
58	MEDP0025	C11H12N2O2	L-Tryptophan	-2.73	down
59	MEDP0041	C6H12N2O3	Ala-Ala	1.08	up
60	MEDP0043	C20H32N6O12S2	Glutathione Oxidized	1.05	up
61	MEDP0044	C10H17N3O6S	Glutathione Reducedform	2.40	up
62	MEDP0066	C11H13NO4	N-Acetyl-L-Tyrosine	-1.19	down
63	MEDP0075	C8H16N4O3	Nα-Acetyl-L-Arginine	-1.31	down
64	MEDP0077	C13H16N2O4	Phenylacetyl-L-Glutamine	1.75	up
65	MEDP0085	C8H11NO	Tyramine	-1.13	down
66	MEDP0101	C9H8O3	P-Coumaric Acid	-1.96	down
67	MEDP0120	C7H8N4O2	Theobromine	-10.55	down

68	MEDP0143	C10H16N2O3S	Biotin	-1.36	down
69	MEDP0149	C10H14N5O6P	2'-Deoxyadenosine-5'-Monophosphate	1.99	up
70	MEDP0152	C10H14N5O7P	3'-Aenylic Acid	2.43	up
71	MEDP0159	C5H5N5	Adenine	-1.18	down
72	MEDP0161	C10H15N5O10P2	Adenosine 5'-Diphosphate	2.49	up
73	MEDP0164	C9H14N3O8P	Cytidine-5-Monophosphate	-1.02	down
74	MEDP0169	C10H14N5O8P	Guanosine-5'-monophosphate	1.49	up
75	MEDP0173	C21H27N7O14P2	Nicotinic Acid Adenine Dinucleotide	1.43	up
76	MEDP0203	C9H8O	Trans-Cinnamaldehyde	-1.55	down
77	MEDP0213	C11H14N2	N-Methyltryptamine	1.07	up
78	MEDP0246	C6H5NO2	Nicotinic Acid	-1.84	down
79	MEDP0284	C12H10O2	1-Naphthylacetic Acid	-2.64	down
80	MEDP0332	C9H8O2	Cinnamic Acid	-2.06	down
81	MEDP0374	C10H12N5O6P	Cyclic Amp	1.63	up
82	MEDP0394	C20H28O3	4-Hydroxyretinoic Acid	-1.03	down
83	MEDP0401	C11H15N5O3S	5'-Deoxy-5'-(Methylthio) Adenosine	1.01	up
84	MEDP0409	C5H10N2O3S	Cys-Gly	1.24	up
85	MEDP0443	C20H23N7O7	10-Formyl-Thf	1.53	up
86	MEDP0507	C9H8O3	2-Hydroxycinnamic acid	-1.96	down
87	MEDP0514	C12H17N4OS+	Thiamine	1.47	up
88	MEDP0519	C6H13NO2	L-Norleucine	-2.52	down
89	MEDP0574	C18H35NO	Oleamide	1.90	up
90	MEDP0618	C12H23NO4	Carnitine 2-methyl-C4	-1.75	down
91	MEDP0663	C5H13N	3-Methyl-1-butylamine	-1.02	down
92	MEDP0685	C4H9NO2S	Methylcysteine	-1.69	down
93	MEDP0849	C7H12N2O4	Nα-Acetyl-L-glutamine	-1.59	down
94	MEDP0883	C6H4N4O2	2,4-Dihydroxypteridine	-1.02	down
95	MEDP0891	C11H13N3O	L-Tryptophanamide	-2.75	down
96	MEDP1002	C7H15NO2	Acetylcholine	1.12	up
97	MEDP1013	C8H14N2O5S	γ-L-Glutamate-Cysteine	2.75	up
98	MEDP1060	C9H7NO	Hydroxyquinoline	-1.68	down
99	MEDP1062	C3H6OS	Thioacetic acid-S-methyl ester	-1.08	down
100	MEDP1073	C6H7N5O	6-O-methylguanine	-1.13	down
101	MEDP1077	C11H15N5O4	2'-O-methyladenosine	2.43	up
102	MEDP1097	C21H38O4	MG(18:2/0:0/0:0)	-1.14	down
103	MEDP1124	C20H32O3	15-HETE	1.08	up
104	MEDP1132	C7H15NO2	3-Carboxypropyltrimethylammonium	1.12	up
105	MEDP1153	C20H32O3	20-HETE	1.08	up
106	MEDP1162	C6H7N5O	1-Methylguanine	-1.13	down
107	MEDP1231	C20H23N7O7	Folic acid	1.53	up
108	MEDP1278	C6H9NOS	4-methyl-5-thiazole ethanol	1.74	up
109	MEDP1280	C15H22N6O5S	S-Adenosyl-L-Methionine	1.15	up
110	MEDP1286	C12H20O2	3,7-Dimethyl-2E, 6-octadienyl acetate	1.31	up
111	MEDP1292	C21H35N7O13P2S	3'-Dephosphocoenzyme A	1.41	up
112	MEDP1298	C21H36O3	Pregnanetriol	1.36	up
113	MEDP1367	C12H16N2O4	Ala-Tyr	-1.22	down
114	MEDP1423	C14H25NO6	Carnitine C7:DC	1.12	up
115	MEDP1428	C15H27NO4	Carnitine C8:1	3.48	up
116	MEDP1430	C14H27NO4	Carnitine C7:0	2.78	up
117	MEDP1431	C14H25NO4	Carnitine C7:1Isomer1	1.68	up
118	MEDP1432	C14H25NO4	Carnitine C7:1	2.08	up
119	MEDP1434	C11H19NO6	Carnitine C4:DC	1.16	up
120	MEDP1438	C13H23NO4	Carnitine C6:1	13.77	up
121	MEDP1440	C12H23NO4	Carnitine C5:0	-1.75	down
122	MEDP1481	C15H14O4	Menadiol Diacetate	1.39	up
123	MEDP1485	C9H9NOS2	2-(2-Benzothiazolylthio)ethanol	-12.44	down
124	MEDP1493	C11H15N5O4	N6-methyladenosine	-1.14	down
125	MEDP1509	C10H18N2O5S	Glu-Met	1.49	up
126	MEDP1511	C10H18N2O5S	Met-Glu	1.49	up
127	MEDP1516	C17H31NO4	Carnitine C10:1 Isomer1	2.08	up
128	MEDP1646	C6H17N3	Caldine	-2.05	down
129	MEDP1647	C8H11NO	2-Amino-1-phenylethanol	-1.03	down
130	MEDP1654	C11H15N5O4	1-Methyladenosine	-1.14	down
131	MEDP1741	C5H14N4	Agmatine	-2.09	down
132	MEDP1748	C6H10N6	Cyromazine	-1.59	down
133	MEDP1759	C22H28O2	Etonogestrel	-12.49	down
134	MEDP1761	C6H13NO5	Glucosamine	-2.15	down
135	MEDP1777	C5H11NO3S	L-Methionine sulfoxide	2.80	up
136	MEDP1786	C11H23N3O2	N1,N8-diacetylspermidine	1.37	up

137	MEDP1820	C9H10Cl2N4	Apraclonidine	11.68	up
138	MEDP1859	C14H19N3O4	Gln-Phe	1.02	up
139	MEDP1889	C12H16N2O4	Ser-Phe	1.48	up
140	MEDP1900	C8H10N4O2	Caffeine	-1.21	down
141	MEDP1907	C18H35NO	(E,Z)-2-Amino-3,14-octadecadien-1-ol	1.90	up
142	MEDP1909	C5H6O2	1,2-Cyclopentanedione	1.26	up
143	MEDP1914	C15H21NO7	N-(1-Deoxy-1-fructosyl)phenylalanine	1.31	up
144	MEDP1961	C15H21N5O8	Ribosyladenosine	-1.52	down
145	MEDP1966	C6H11NO2S	S-Allyl-L-cysteine	-1.79	down
146	MEDP1988	CH4N2O2	Hydroxyurea	-1.79	down
147	MEDP2027	C6H13NO5	2-Amino-2-deoxymannose	-2.15	down
148	MEDP2038	C10H13NO4	3-O-Methyldopa	-1.14	down
149	MW0000069	C18H19NO5	1-O-Acetyllycorine	1.55	up
150	MW0001484	C23H34N2O4S	11-(Dansylamino)undecanoic acid	1.23	up
151	MW0005864	C15H13NO2	7-Hydroxy-2-acetylaminofluorene	-1.46	down
152	MW0006061	C14H8O2	Anthraquinone	9.76	up
153	MW0006138	C10H12N2O3S	Bentazone	16.56	up
154	MW0006838	C10H14O3	Dihydroconiferyl alcohol	2.58	up
155	MW0007009	C11H15NO4S	Ethiofencarb sulfone	16.19	up
156	MW0007370	C19H15ClN2O2	Inabenfide	15.76	up
157	MW0008652	C26H28N4O2	N-[2-(4-Oxo-1-phenyl-1,3,8-triazaspiro[4.5]decan-8-yl)ethyl]naphthalene-2-carboxamide	1.77	up
158	MW0009371	C9H13ClNO3P	Phaclofen	15.35	up
159	MW0009788	C12H14N4O4S	Sulfadoxin	-1.23	down
160	MW0009795	C9H10N4O2S2	Sulfamethizole	-2.18	down
161	MW0009889	C13H11Cl2F4N3C	Tetraconazole	17.75	up
162	MW0010819	C18H32O5	(10E,15Z)-9,12,13-Trihydroxyoctadeca-10,15-dienoic acid	1.59	up
163	MW0011898	C19H36O5	1,2-Dioctanoyl-sn-glycerol	2.79	up
164	MW0012046	C20H34O5	11,12,15-THETA	1.94	up
165	MW0012099	C20H30O6	11-dehydro-TXB3	2.13	up
166	MW0012153	C20H32O3	11S-Hydroxy-5Z,8Z,12E,14Z-eicosatetraenoic acid	-1.61	down
167	MW0012180	C20H32O3	12(R)-HETE	1.05	up
168	MW0012181	C20H32O4	12(R)-HPETE	1.87	up
169	MW0012182	C20H32O4	12(S),20-DiHETE	2.14	up
170	MW0012337	C28H42O3	13'-Hydroxy-gamma-tocotrienol	-1.13	down
171	MW0012501	C20H36O4	15S-Hydroperoxy-11Z,13E-eicosadienoic acid	-1.32	down
172	MW0012515	C22H36O6	16,16-dimethyl-6-keto Prostaglandin E1	2.16	up
173	MW0012521	C22H36O5	16,16-dimethyl-PGE2	1.39	up
174	MW0012645	C23H30O5	17-phenyl trinor 8-iso Prostaglandin E2	1.75	up
175	MW0012742	C22H28O3	19-Norethindrone acetate	1.23	up
176	MW0012778	C30H62NO7P	1-Behenoyl-2-hydroxy-sn-glycero-3-phosphocholine	-1.27	down
177	MW0013121	C21H25F3O6	2,3-Dinorfluprostenol	1.88	up
178	MW0013139	C19H34O4	2,4-Dihydroxyheptadec-16-ynyl acetate	1.07	up
179	MW0013261	C21H30O4	21-Hydroxy-5b-pregnane-3,11,20-trione	-1.16	down
180	MW0013368	C29H50O6	28-Homobrassinolide	1.03	up
181	MW0013622	C9H8O7	2-oxo-3-(2,3,4,5-tetrahydroxyphenyl)propanoic acid	11.51	up
182	MW0013853	C30H40O7	3,7,11,15,23-Pentaoxolanost-8-en-26-oic acid	2.51	up
183	MW0014140	C7H12O5	3-Isopropylmalic acid	-1.00	down
184	MW0014357	C13H22O3	4,5-Dihydrovomifoliol	1.67	up
185	MW0014593	C21H34O3	5(6)-Epoxy-8Z,11Z,14Z-eicosatrienoic acid, methyl ester	1.03	up
186	MW0014720	C27H48O5	5beta-cholestan-3alpha,7alpha,12alpha,24(S),27-pentol	1.95	up
187	MW0014839	C20H32O4	5S-HpETE	1.22	up
188	MW0014866	C19H30O5S	5α-Androstan-17β-ol-3-one sulfate	1.73	up
189	MW0015106	C15H28O4	7(14)-Bisabolene-2,3,10,11-tetrol	1.22	up
190	MW0015228	C21H28O3	7-Oxocallitrisic acid, methyl ester	2.18	up
191	MW0015486	C18H28O3	9-OxoOTrE	1.38	up
192	MW0015731	C10H18O	alpha-TERPINEOL	1.91	up
193	MW0015755	C28H35FO7	Amcinonide	-1.36	down
194	MW0015833	C32H46N2O8	Anthranoyllycoctonine	16.03	up
195	MW0015892	C12H16O7	Arbutin	-1.59	down
196	MW0016017	C17H32O3	Avocadyne	2.35	up
197	MW0016018	C19H34O4	Avocadyne 1-acetate	1.28	up
198	MW0016120	C30H46O4	beta-Glycyrrhetinic acid	1.12	up
199	MW0016359	C40H56O3	Capsanthin	-1.46	down
200	MW0017075	C27H42O	Cholesta-4,6-dien-3-one	-1.16	down
201	MW0017171	C17H32O2	cis-7-Hexadecenoic acid methyl ester	1.02	up
202	MW0017210	C22H40O11	Citronellyl beta-sophoroside	-1.86	down
203	MW0049036	C21H36O5	Cortol	1.11	up
204	MW0049211	C27H40O2	delta-Tocotrienol	-2.03	down

205	MW0049213	C30H44O2	Demethylphylloquinone	-1.12	down
206	MW0052562	C19H30O2	Epiandrosterone	1.10	up
207	MW0052767	C19H28O2	Etiocholanedione	1.03	up
208	MW0052949	C16H24O2	Furanofukinin	1.36	up
209	MW0053478	C16H22O10	Geniposidic acid	1.75	up
210	MW0054021	C20H28O5	Ingenol	1.07	up
211	MW0054215	C20H34O4	Kirenol	-1.13	down
212	MW0054284	C25H39NO4	Latanoprost ethyl amide	-1.34	down
213	MW0054375	C20H36O2	Linoleic acid ethyl ester	1.84	up
214	MW0054786	C16H28O3	Methoprene acid	1.28	up
215	MW0054787	C19H34O3	Methoprene;(E,E)-1-Methylethyl 11-methoxy-3,7,11-trimethyl-2,4-dodecadienoate	1.16	up
216	MW0055179	C21H39NO6	Myriocin	2.49	up
217	MW0055321	C15H20O7	Nivalenol	-1.25	down
218	MW0055427	C16H23N5O6	O-beta-D-Glucosyl-trans-zeatin	-3.59	down
219	MW0062074	C15H13N3O4S	Piroxicam	15.73	up
220	MW0062131	C28H44O5	Polyporusterone D	1.05	up
221	MW0062134	C28H44O5	Polyporusterone G	1.34	up
222	MW0063357	C17H32O4	Roccellic acid	1.04	up
223	MW0063530	C27H43NO3	Sipeimine	-1.04	down
224	MW0063652	C21H32N2O	Stanozolol	1.23	up
225	MW0063660	C18H32O2	Stearolic acid	1.35	up
226	MW0063717	C25H34F2O5	Tafluprost	1.44	up
227	MW0102839	C20H28O3	Totarol-19-carboxylic acid	1.23	up
228	MW0102970	C20H34O5	Trioxilin A3	1.08	up
229	MW0103146	C23H24O8	Wortmannin	1.06	up
230	MW0103443	C12H16N5O8P	Acetyl adenylate	1.51	up
231	MW0103574	C9H11F2N3O4	Gemcitabine	1.42	up
232	MW0103583	C16H25N5O16P2	Guanosine diphosphate mannose	2.72	up
233	MW0104066	C11H14O6S	[2,6-dihydroxy-3-(3-methylbut-2-en-1-yl)phenyl]oxidanesulfonic acid	-1.10	down
234	MW0104210	C18H28O6	11-dehydro-2,3-dinor Thromboxane B2	-1.34	down
235	MW0104373	C11H18O4	2,2'-(3-methylcyclohexane-1,1-diyl)diacetic acid	1.25	up
236	MW0105048	C7H14O3	3-Hydroxyisoheptanoic acid	2.20	up
237	MW0105424	C8H15NO3	Acetylleucine	14.32	up
238	MW0105481	C13H21N3O6	Ala-Pro-Glu	1.13	up
239	MW0105667	C16H31N7O6	Arg-Lys-Asp	5.05	up
240	MW0105731	C17H24N6O3	Arginyl-Tryptophan	23.63	up
241	MW0105881	C19H28N4O7	Asp-Tyr-Lys	21.34	up
242	MW0106000	C14H20Cl2N2O6	Bactobolin	18.07	up
243	MW0107571	C15H22N2O3	Isoleucyl-Phenylalanine	1.07	up
244	MW0107823	C31H49N3O9S	Leukotriene C4 methyl ester	1.41	up
245	MW0107992	C10H20N4O4	Lys-Asn	1.06	up
246	MW0108302	C2H6N2O	Methylurea	-1.81	down
247	MW0108638	C7H11NO4	N-Acetyl-L-glutamate 5-semialdehyde	-1.17	down
248	MW0108713	C8H13NO3	N-Butyryl-L-homoserine lactone	17.45	up
249	MW0108798	C20H37NO3	N-Hexadecanoyl-L-homoserine lactone	1.62	up
250	MW0109039	C22H42N4O8S2	Pantethine	-4.99	down
251	MW0109048	C20H21N5O6	Pemetrexed	16.53	up
252	MW0109133	C13H18N2O4	Phe-Thr	-1.01	down
253	MW0109212	C12H16N2O4	Phenylalanyl-Serine	1.17	up
254	MW0109278	C22H24O10	Piperenol C	1.52	up
255	MW0109322	C13H23N3O4	Pro-Leu-Gly	2.42	up
256	MW0109553	C17H22N4O8S	S-(4-Nitrobenzyl)glutathione	9.99	up
257	MW0109750	C11H13NO3S	S-Phenylmercapturic acid	15.53	up
258	MW0110177	C21H37NO5	Tumonoic Acid F	-1.35	down
259	MW0110284	C11H14N2O4	Tyrosyl-Glycine	17.22	up
260	MW0110293	C12H16N2O5	Tyrosyl-Serine	-4.30	down
261	MW0111058	C9H10ClN5O2	Imidacloprid	17.53	up
262	MW0111083	C12H21N	Memantine	17.86	up
263	MW0111335	C6H18N4	Triethylenetetramine	1.86	up
264	MW0114245	C16H20N4O6	Diaziquone	-1.01	down
265	MW0114564	C2H4O2	Glycolaldehyde	-1.46	down
266	MW0114889	C16H30O	Muscone	1.27	up
267	MW0115122	C12H14O7	Phenyl glucuronide	13.42	up
268	MW0115397	C8H14O3	trans-4-Hydroxycyclohexylacetic acid	1.27	up
269	MW0118357	C8H12O	2-Butylfuran	1.11	up
270	MW0118565	C10H20O3	2-Hexyl-1,3-dioxan-5-ol	-2.20	down
271	MW0119688	C17H29N4O8P2S	3-Methyl-1-hydroxybutyl-ThPP	15.02	up
272	MW0120025	C20H14N2O5	4,5-Diaminofluorescein	1.06	up

273	MW0120648	C13H8N2O3	4-Oxo-1H-1,10-phenanthroline-3-carboxylic acid	9.02	up
274	MW0120912	C19H32O4	5-(Tetradecyloxy)-2-furoic acid	1.14	up
275	MW0121482	C9H6FNO2	5-Fluoroindole-2-carboxylic acid	-2.38	down
276	MW0122305	C9H9N5O3	6-Succinoaminopurine	14.92	up
277	MW0122692	C8H9N5O4	9-Carboxymethoxymethylguanine	17.66	up
278	MW0122713	C24H34N2O2	9-Octadecyn-1-one, 1-oxazolo[4,5-b]pyridin-2-yl-	1.30	up
279	MW0122830	C10H13N3O2S	Aminoalbendazole sulfone	21.43	up
280	MW0122849	C22H39NO5	AMP-Deoxynojirimycin	-1.03	down
281	MW0122947	C28H31FN4O	Astemizole	7.24	up
282	MW0123463	C15H23NO4	Cycloheximide	-1.98	down
283	MW0123586	C12H14N4O8	D-Glucose, 2-deoxy-2-[(7-nitro-2,1,3-benzoxadiazol-4-yl)amino]-	17.37	up
284	MW0123814	C17H26N4O	Emedastine	-3.08	down
285	MW0124112	C22H28N2O	Fentanyl	1.41	up
286	MW0124326	C20H24N2O2S	Hycanthone	-1.24	down
287	MW0124694	C20H26N4O	Lisuride	16.87	up
288	MW0124733	C23H22O6	Macluraxanthone	1.20	up
289	MW0124785	C16H21ClN4	Mepiprazole	1.34	up
290	MW0125116	C11H11NO4	Methyl 5-hydroxyoxindole-3-acetate	12.71	up
291	MW0125186	C12H9N3O	Milrinone	20.92	up
292	MW0125187	C17H22N4O	Minaprine	1.24	up
293	MW0125621	C10H10N2O4S	N-acetyl zonisamide	14.38	up
294	MW0125832	C8H6N4O5	Nitrofurantoin	18.24	up
295	MW0126047	C27H33NO4	Paxilline	1.24	up
296	MW0126063	C37H44ClNO6	Penitrem A	-1.49	down
297	MW0126093	C13H8N2O2	Phenazine-1-carboxylic acid	20.80	up
298	MW0126181	C23H29NO3	Piperundecalidine	-2.58	down
299	MW0126274	C12H17N2O4P	Psilocybine	16.15	up
300	MW0126665	C26H45NO4	Tert-butyl (4S)-4-hexadec-2-ynoyl-2,2-dimethyl-1,3-oxazolidine-3-carboxylate	1.36	up
301	MW0127559	C9H19NOS	S-Ethyl dipropylthiocarbamate	-2.05	down
302	MW0127590	C15H14O6	(-)-Catechin	15.60	up
303	MW0128936	C14H14O7S	{[1-(7-hydroxy-2-oxo-2H-chromen-8-yl)-3-methylbut-3-en-2-yl]oxy}sulfonic acid	-2.11	down
304	MW0130161	C33H40O22	2-(3,4-Dihydroxyphenyl)-5,7-dihydroxy-4-oxo-4H-chromen-3-yl hexopyranosyl-(1->2)hexopyranosyl-(1->2)hexopyranoside	1.36	up
305	MW0131039	C26H29NO3	3-(2-{4-[2-(dimethylamino)ethoxy]phenyl}-3-ethyl-3-phenyloxiran-2-yl)phenol	1.30	up
306	MW0132539	C12H16O5	3,4,5-Trimethoxydihydrocinnamic acid	1.06	up
307	MW0133090	C14H18O4	3-hydroxy-3-[4-hydroxy-3-(3-methylbut-2-en-1-yl)phenyl]propanoic acid	-2.45	down
308	MW0133326	C26H30O13	4-(7-Hydroxy-4-oxo-3,4-dihydro-2H-chromen-2-yl)phenyl 2-O-(3,4-dihydroxy-4-(hydroxymethyl)tetrahydrofuran-2-yl)hexopyranoside	13.15	up
309	MW0134121	C26H28O13	5,7-Dihydroxy-2-phenyl-6-[3,4,5-trihydroxy-6-(hydroxymethyl)oxan-2-yl]-8-(3,4,5-trihydroxyoxan-2-yl)chromen-4-one	15.81	up
310	MW0136894	C24H32O5	7-[(2,6-Dihydroxy-2,5,5,8a-tetramethyldecalin-1-yl)methoxy]coumarin	1.28	up
311	MW0138538	C20H24O10	Isorutarin	1.31	up
312	MW0138859	C29H35O17	Malvidin 3,5-diglucoside cation	13.05	up
313	MW0139214	C23H26O9	Osmanthuside A	1.47	up
314	MW0139227	C14H12O4	Oxyresveratrol	19.90	up
315	MW0139677	C16H18O9	Scopolin	17.83	up
316	MW0139797	C23H22O7	Tephrosin	-1.18	down
317	MW0139900	C9H6O3	Umbelliferone	18.30	up
318	MW0140187	C15H26O4	(10S)-Juvenile hormone III acid diol	1.57	up
319	MW0140188	C16H28O4	(10S)-Juvenile hormone III diol	2.20	up
320	MW0141034	C11H14NO4P	1-(Indol-3-yl)propanol 3-phosphate;Indolepropanol phosphate	15.33	up
321	MW0141219	C17H28O4	10-Deoxymethynolide	-1.23	down
322	MW0141353	C20H36O5	13,14-dihydro PGF2	-17.17	down
323	MW0141363	C16H28O5	13,14-dihydro-15-keto-tetranor PGF1	-1.43	down
324	MW0141364	C16H26O5	13,14-dihydro-15-keto-tetranor Prostaglandin D2	1.66	up
325	MW0141407	C20H34O6	15(R),19(R)-hydroxy PGF2	-2.49	down
326	MW0141455	C37H48N2O7	16,16-dimethyl Prostaglandin E2 p-(p-acetamidobenzamido) phenyl ester	13.72	up
327	MW0141638	C26H39NO6	17-phenyl trinor Prostaglandin F2 serinol amide	11.49	up
328	MW0141666	C20H36O6	19(R)-hydroxy-PGF1	-1.57	down
329	MW0142012	C16H30O4	2,3-Dihydroxycyclopentaneundecanoic acid	1.09	up
330	MW0142162	C22H31FO4	21-Fluoro-11beta,17-dihydroxy-6alpha-methylpregn-4-ene-3,20-dione;21-Fluoro-11beta,17-dihydroxy-6alpha-methylprogesterone	2.16	up
331	MW0142453	C17H32O3	2-Methoxy-5Z-hexadecenoic acid;2-Methoxy-5Z-hexadecenoate;(5Z)-2-Methoxyhexadecenoic acid	1.91	up

332	MW0142680	C41H62O2	3',4'-Dihydrorhodovibrin	1.33	up
333	MW0142720	C9H9N3O7	3,5-Dinitro-L-tyrosine	17.43	up
334	MW0142903	C8H14O8	3-Deoxy-D-manno-octulosonate;Ketodeoxyoctonate;KDO;2-Dehydro-3-deoxy-D-octonate;3-Deoxy-D-manno-2-octulosonate;3-Deoxyoctulosonic acid	-1.50	down
335	MW0142905	C18H24O	3-Deoxyestradiol;3-Deoxy-17beta-estradiol	1.18	up
336	MW0143193	C20H28F2O2	4,4-Difluoro-17beta-hydroxy-17alpha-methyl-androst-5-en-3-one	1.00	up
337	MW0143273	C15H14ClN	4-Chloro-[2-(4-Pyridinyl)-1-butenyl]phenol;4-[1-[(4-Chlorophenyl)methylene]propyl]pyridine	-1.29	down
338	MW0143480	C10H9NO7	5-(3'-Carboxy-3'-oxopropyl)-4,6-dihydroxypicolinate;5-(gamma-Carboxy-gamma-oxopropyl)-4,6-dihydroxypicolinate	13.60	up
339	MW0143482	C21H29N5O2	5-(3-Hydroxy-4-phenylbut-1-en-1-yl)-1-[6-(2H-tetrazol-5-yl)hexyl]pyrrolidin-2-one	1.56	up
340	MW0143662	C6H12O4S	5-Methylthio-D-ribose;S-Methyl-5-thio-D-ribose	13.44	up
341	MW0143816	C21H38O6	6-Deoxyerythronolide B	1.20	up
342	MW0143834	C27H40O2	6-Geranylgeranyl-2-methylbenzene-1,4-diol	2.54	up
343	MW0143850	C21H25N3O3	6-Hydroxytryprostatin B;Desmethyltryprostatin A	-2.11	down
344	MW0143867	C20H25NO4	6Naltrexol	2.15	up
345	MW0143901	C27H46O2	7 -Hydroxycholesterol	-1.18	down
346	MW0144115	C18H34O4	9,10-Epoxy-18-hydroxystearate	1.53	up
347	MW0144117	C21H36O4	9,11-methane-epoxy PGF1	1.62	up
348	MW0144423	C17H12O8	Aflatoxin-M1-8,9-epoxide	1.74	up
349	MW0144506	C16H27N5O8	Ala-Asn-Val-Asp	-1.02	down
350	MW0144508	C19H36N10O7	Ala-Asp-Arg-Arg	15.49	up
351	MW0144519	C15H24N4O10	Ala-Asp-Ser-Glu	-1.08	down
352	MW0144661	C22H43N9O6	Ala-Lys-Gly-Arg-Val	-1.49	down
353	MW0144747	C19H37N5O6	Ala-Thr-Ile-Lys	1.62	up
354	MW0144760	C19H24N4O6	Ala-Trp-Glu	-1.65	down
355	MW0144790	C16H27N5O8	Ala-Val-Asn-Asp	12.05	up
356	MW0145230	C12H17O10P	Arbutin 6-phosphate;Arbutin-6P	1.84	up
357	MW0145298	C28H44N8O10	Arg-Asp-Leu-Tyr-Ser	4.34	up
358	MW0145343	C21H38N10O9	Arg-Glu-Asp-Arg	-2.41	down
359	MW0145488	C20H30N6O5	Arg-Pro-Tyr	-1.51	down
360	MW0145509	C19H38N10O6	Arg-Thr-Ala-Arg	2.41	up
361	MW0145701	C20H32N6O11	Asn-Asp-Gly-Val-Glu	-1.00	down
362	MW0145796	C25H40N6O6	Asn-Ile-Phe-Lys	2.19	up
363	MW0145924	C23H34N8O9	Asn-Tyr-Arg-Asp	12.89	up
364	MW0146182	C27H50N10O10	Asp-Lys-Arg-Glu-Lys	18.27	up
365	MW0146246	C24H39N5O11	Asp-Pro-Thr-Ile-Glu	-1.15	down
366	MW0146971	C27H40O3	Calcipotriol	1.04	up
367	MW0147552	C20H34O	cis-Abienol;(12Z)-Labda-12,14-dien-8alpha-ol	1.26	up
368	MW0148190	C23H26O6	Decarboxy-Norlobaric Acid	2.32	up
369	MW0148196	C19H32O4	Decylubiquinol;6-Decylubiquinol;2-Decyl-5,6-dimethoxy-3-methyl-1,4-benzenediol	1.92	up
370	MW0148197	C19H30O4	Decylubiquinone;6-Decylubiquinone;2,3-Dimethoxy-5-methyl-6-decyl-1,4-benzoquinone	1.67	up
371	MW0148540	C24H32N4O2	Dimethamine	1.42	up
372	MW0149444	C9H16N2O5	gamma-L-Glutamyl-L-2-aminobutyrate	2.14	up
373	MW0149527	C20H41N5O7	Gentamicin C2a	17.30	up
374	MW0149564	C20H28O3	Gibberellin A12 aldehyde	-2.16	down
375	MW0149739	C22H40N6O8	Gln-Leu-Glu-Lys	-1.01	down
376	MW0150397	C23H42N10O8	Gly-Leu-Arg-Asn-Gln	10.97	up
377	MW0150398	C28H46N8O6	Gly-Leu-Arg-Val-Phe	3.87	up
378	MW0150995	C32H50N8O7	His-Leu-Lys-Tyr-Val	1.31	up
379	MW0151023	C15H24N6O5S1	His-Met-Asn	1.06	up
380	MW0151266	C41H60O3	Hydroxyspheroidenone	1.18	up
381	MW0151323	C20H36N8O8	Ile-Arg-Asn-Asp	6.20	up
382	MW0151417	C23H39N7O7	Ile-Glu-His-Lys	1.14	up
383	MW0151444	C20H32N6O7	Ile-His-Ala-Glu	-1.17	down
384	MW0151465	C24H46N8O8	Ile-Ile-Arg-Ser-Ser	14.71	up
385	MW0151597	C20H32N6O8	Ile-Thr-His-Asp	1.09	up
386	MW0151648	C30H48N6O7	Ile-Val-Ile-Phe-Asn	-1.09	down
387	MW0151922	C22H18O7	Justicidin A	1.26	up
388	MW0151979	C27H29NO	JWH182	1.67	up
389	MW0152006	C17H20O4	Karwinaphthol B	1.58	up
390	MW0152165	C8H16O4	L-Cladinose	1.08	up
391	MW0152260	C25H43N7O10	Leu-Asp-Gln-Gln-Val	-1.07	down
392	MW0152383	C20H36N4O8	Leu-Ile-Ser-Glu	2.00	up
393	MW0152486	C20H37N5O8	Leu-Ser-Glu-Lys	12.88	up

394	MW0152632	C16H22N2O6	Leu-Val-OH	2.10	up
395	MW0152852	C14H28N4O4S1	Lys-Ala-Met	-1.04	down
396	MW0152962	C26H42N6O7	Lys-Glu-Phe-Lys	1.59	up
397	MW0153118	C16H30N4O4S1	Lys-Pro-Met	1.89	up
398	MW0153180	C26H41N5O8	Lys-Tyr-Ile-Glu	14.58	up
399	MW0153230	C17H26N4O6	Lys-Lys-OH	1.02	up
400	MW0153611	C20H32N4O4S1	Met-Lys-Phe	1.52	up
401	MW0153637	C27H39N5O11S	Met-Phe-Thr-Glu-Asp	-1.79	down
402	MW0154133	C8H21N5	N1-(3-Aminopropyl)agmatine;N1-Aminopropylagmatine	1.36	up
403	MW0154302	C20H32O5	Narbonolide	1.13	up
404	MW0154332	C22H39NO3	N-cis-octadec-9Z-enoyl-L-Homoserine lactone	1.58	up
405	MW0154695	C18H32O4	Octadec-9-ene-1,18-dioic-acid	-1.81	down
406	MW0155155	C26H43N9O6	Phe-Gln-Arg-Lys	5.48	up
407	MW0155408	C26H39N9O5	Phe-Val-His-Arg	-2.16	down
408	MW0155760	C22H38N6O9	Pro-Ala-Asp-Lys-Thr	16.34	up
409	MW0155777	C16H29N7O5	Pro-Arg-Gln	-1.34	down
410	MW0155862	C15H25N3O6	Pro-Glu-Val	5.14	up
411	MW0155922	C15H26N4O6	Pro-Lys-Asp	-1.20	down
412	MW0156004	C15H23N5O5	Pro-Thr-His	-1.99	down
413	MW0156128	C35H60N4O6S	Prostaglandin F2 -biotin	1.13	up
414	MW0156198	C21H40O16P2	PtdIns-(5)-P1(1,2-dihexanoyl)(sodium salt)	14.45	up
415	MW0156675	C17H29N7O10	Ser-Asp-Arg-Asp	1.71	up
416	MW0156712	C20H38N6O7	Ser-Gln-Leu-Lys	1.02	up
417	MW0156720	C19H36N6O7	Ser-Gln-Val-Lys	-1.02	down
418	MW0156940	C32H38N6O9	Ser-Trp-Asp-Pro-Phe	1.37	up
419	MW0157200	C42H58N2O	Staphidine	1.84	up
420	MW0157549	C22H40N8O9	Thr-Arg-Gly-Glu-Val	1.01	up
421	MW0157680	C19H33N5O9	Thr-Ile-Gln-Asp	14.54	up
422	MW0157727	C16H28N6O5	Thr-Lys-His	-2.67	down
423	MW0158067	C20H34O5	Trioxilin B3;(5Z,8Z,14Z)-(11R,12R)-10,11,12-Trihydroxyeicosa-5,8,14-trienoic acid;(5Z,8Z,14Z)-(11R,12R)-10,11,12-Trihydroxyicosa-5,8,14-trienoic acid	1.94	up
424	MW0158136	C25H29N5O5	Trp-Gln-Phe	-2.82	down
425	MW0158236	C17H22N4O5	Trp-Ser-Ala	16.30	up
426	MW0158293	C21H21N3O6	Trp-Abu-OH	-2.00	down
427	MW0158294	C20H19N3O6	Trp-Ala-OH	15.35	up
428	MW0158378	C21H34N6O5	Tyr-Arg-Leu	15.37	up
429	MW0158481	C21H25N7O5	Tyr-His-His	1.19	up
430	MW0158562	C22H26N4O6	Tyr-Phe-Asn	1.61	up
431	MW0158652	C24H31N3O6	Tyr-Tyr-Leu	1.33	up
432	MW0158670	C29H48N6O7	Tyr-Val-Lys-Ala-Leu	1.19	up
433	MW0158691	C21H24N2O7	Tyr-Ile-OH	-1.31	down
434	MW0158829	C2H4N2O3	Urea-1-carboxylate;Allophanate;Allophanic acid	-2.58	down
435	MW0158878	C21H30N4O7	Val-Ala-Phe-Asp	4.88	up
436	MW0158961	C19H36N8O6	Val-Gln-Ala-Arg	1.04	up
437	MW0158974	C23H33N5O8	Val-Gln-Phe-Asp	1.02	up
438	MW0159042	C21H38N4O7	Val-Ile-Leu-Asp	1.63	up
439	MW0159058	C29H43N5O10	Val-Leu-Asp-Phe-Glu	1.25	up
440	MW0159068	C23H43N5O7S	Val-Leu-Leu-Ser-Cys	14.61	up
441	MW0159124	C24H37N5O7	Val-Phe-Lys-Asp	13.41	up
442	MW0159212	C30H44N8O8	Val-Tyr-Gln-His-Val	13.34	up
443	MW0159911	C19H28O2	17-hydroxy-10,13-dimethyl-1,2,6,7,8,9,11,12,14,15,16,17-dodecahydrocyclopenta[a]phenanthren-3-one	1.15	up
444	MW0160134	C10H12N2O4S	[3-(2-aminoethyl)-1H-indol-5-yl] hydrogen sulfate	19.13	up
445	MW0160174	C20H30O2	(4aS,4bS,7S,9S,10aS)-7-ethenyl-9-hydroxy-1,1,4a,7-tetramethyl-3,4,4b,5,6,9,10,10a-octahydrophenanthren-2-one	1.07	up
446	MW0160197	C20H26N2O2	(9R,10S,12S,13S,14R,16S,18R)-13-ethyl-8-methyl-8,15-diazahexacyclo[14.2.1.01,9.02,7.010,15.012,17]nonadeca-2,4,6-triene-14,18-diol	-1.11	down
447	MW0160293	C6H14O12P2	[(2S,3S,4S,5R)-2,3,4-trihydroxy-5-(phosphonooxymethyl)oxolan-2-yl]methyl dihydrogen phosphate	2.26	up
448	MW0161410	C40H54O2	3-[(1E,3E,5E,7E,9E,11E,13E,15E,17E)-18-(3-hydroxy-2,6,6-trimethylcyclohexen-1-yl)-3,7,12,16-tetramethyloctadeca-1,3,5,7,9,11,13,15,17-nonaenyl]-2,4,4-trimethylcyclohex-2-en-1-one	1.73	up
449	MW0161563	C10H8N2O	2-(1H-indol-3-yl)acetonitrile oxide	20.74	up
450	MW0161655	C26H31N3O3	(3S,8aS)-3-[[7,7-dimethyl-2-(2-methylbut-3-en-2-yl)-1H-pyrano[2,3-g]indol-3-yl]methyl]-2,3,6,7,8,8a-hexahydropyrrolo[1,2-a]pyrazine-1,4-dione	-1.01	down

451	MW0162029	C20H32O	[(1S,4S,5R,9S,10R,13S)-5,9-dimethyl-14-methylidene-5-tetracyclo[11.2.1.01,10.04,9]hexadecanyl]methanol	1.58	up
452	MW0162139	C16H29N2O14P	[(2R,3S,4R,5R,6R)-5-acetamido-6-[(2R,3S,4R,5R,6R)-5-acetamido-4,6-dihydroxy-2-(hydroxymethyl)oxan-3-yl]oxy-3,4-dihydroxyoxan-2-yl]methyl dihydrogen phosphate	-1.13	down
453	MW0164464	C22H24N2O8	(4S,4aS,5aS,6S,12aR)-2-carbamoyl-4-(dimethylazaniumyl)-6,10,11,12a-tetrahydroxy-6-methyl-3,12-dioxo-4,4a,5,5a-tetrahydrotetracen-1-olate	16.43	up
454	MW0164477	C20H30O3	(5Z,8Z,10E,12E)-13-[(2S,3S)-3-pentyloxiran-2-yl]trideca-5,8,10,12-tetraenoic acid	1.42	up
455	MW0164611	C21H32O5	(3R,5R,8S,9S,10S,14S)-3,11-dihydroxy-17-(2-hydroxyacetyl)-10-methyl-2,3,4,5,6,7,8,9,11,12,14,15,16,17-tetradecahydro-1H-cyclopenta[a]phenanthrene-13-carbaldehyde	-1.16	down
456	MW0165684	C20H32O2	(3R,10R,11S)-14-(hydroxymethyl)-3,10-dimethyl-6-propan-2-yltricyclo[9.3.0.03,7]tetradeca-1(14),6-dien-9-ol	-1.71	down
457	MW0165706	C16H25N5O6	(2R,3S,4S,5R,6S)-2-(hydroxymethyl)-6-[(2S)-2-methyl-4-(7H-purin-6-ylamino)butoxy]oxane-3,4,5-triol	-1.68	down
458	MW0166680	C22H24N2O9	(4S,4aR,5S,5aR,6S,12aR)-2-carbamoyl-4-(dimethylazaniumyl)-5,6,10,11,12a-pentahydroxy-6-methyl-3,12-dioxo-4,4a,5,5a-tetrahydrotetracen-1-olate	15.08	up
459	MW0166718	C22H40O12	[(2R,3S,4R,5R,6R)-6-[(2S,3S,4S,5R)-3,4-dihydroxy-2,5-bis(hydroxymethyl)oxolan-2-yl]oxy-4,5-dihydroxy-2-(hydroxymethyl)oxan-3-yl] decanoate	-1.20	down
460	MW0166724	C16H28O12	[(2R,3S,4R,5R,6R)-6-[(2S,3S,4S,5R)-3,4-dihydroxy-2,5-bis(hydroxymethyl)oxolan-2-yl]oxy-4,5-dihydroxy-2-(hydroxymethyl)oxan-3-yl] 2-methylpropanoate	14.88	up
461	MW0167791	C20H30O2	(2S,4aS,4bR,8S,8aR,10aS)-7-ethenyl-2-hydroxy-1,1,4a,8-tetramethyl-4,4b,5,8,8a,9,10,10a-octahydro-2H-phenanthren-3-one	-1.26	down
462	MW0168220	C20H34O4	Aphidicolin	1.31	up
463	MW0168376	C45H73NO15	Solanine; (2S,3R,4R,5R,6S)-2-[(2R,3R,4S,5S,6R)-5-Hydroxy-6-(hydroxymethyl)-2-[[(1S,2S,7S,10R,11S,14S,15R,16S,17S,20S,23S)-10,14,16,20-tetramethyl-22-azahexacyclo[12.10.0.02,11.05,10.015,23.017,22]tetracos-4-en-7-yl]oxy]-4-[(2S,3R,4S,5S,6R)-3,4,5-trihydroxy-6-(hydroxymethyl)oxan-2-yl]oxyoxan-3-yl]oxy-6-methyloxane-	20.28	up
464	MW0168446	C17H30O3	12(S)-hydroxy-16-Heptadecynoic Acid	1.38	up
465	MW0168860	C14H22BrN3O2	BROMOPRIDE	20.89	up
466	MW0168875	C18H26O2	C18H26O2; PlaSMA ID-1283	1.43	up
