# Supplementary material for: Transcriptomic and metabolomic analyses reveal the antifungal mechanism of the compound phenazine-1-carboxamide on Rhizoctonia solani AG1IA
Source: Front Plant Sci. 2022 Nov 22;13:1041733. doi: 10.3389/fpls.2022.1041733 (PMC9722969; doi:10.3389/fpls.2022.1041733)
Supplement: Supplementary file 5 [file DataSheet_5.pdf]

Supplementary Table 5 Combined analysis of transcriptome and metabolome extremely significantly different gene expression and related metabolites content levels.

Num	Pathway	Difference gene expression level		Related metabolite content level		Correlation coefficient
		Gene Name	Log2FC	Metabolite Name	Fold_Change	
1	Arachidonic acid metabolism	leukotriene-A4 hydrolase (AG1IA_06600)	1.75	Prostaglandin B2 (MEDN1429)	1.12	0.907201175
		leukotriene-A4 hydrolase (AG1IA_06600)	1.75	15-HETE (MEDP1124)	1.08	0.965764247
		leukotriene-A4 hydrolase (AG1IA_06600)	1.75	20-HETE (MEDP1153)	1.08	0.965764247
		leukotriene-A4 hydrolase (AG1IA_06600)	1.75	11,12,15-THETA (MW0012046)	1.94	0.910435399
		leukotriene-A4 hydrolase (AG1IA_06600)	1.75	12(R)-HETE (MW0012180)	1.05	0.952181549
		leukotriene-A4 hydrolase (AG1IA_06600)	1.75	12(R)-HPETE (MW0012181)	1.87	0.928954293
		leukotriene-A4 hydrolase (AG1IA_06600)	1.75	5S-HpETE (MW0014839)	1.22	0.962172101
		leukotriene-A4 hydrolase (AG1IA_06600)	1.75	Trioxilin A3 (MW0102970)	1.08	0.931673262
		alpha/beta hydrolase family domain-containing protein (AG1IA_02292)	-1.92	15-HETE (MEDP1124)	1.08	-0.954464957
		alpha/beta hydrolase family domain-containing protein (AG1IA_02292)	-1.92	20-HETE (MEDP1153)	1.08	-0.954464957
		alpha/beta hydrolase family domain-containing protein (AG1IA_02292)	-1.92	12(R)-HETE (MW0012180)	1.05	-0.966451651
		alpha/beta hydrolase family domain-containing protein (AG1IA_02292)	-1.92	Trioxilin A3 (MW0102970)	1.08	-0.972656648
		fatty acid oxygenase (AG1IA_01703)	-1.73	15-HETE (MEDP1124)	1.08	-0.952116108
		fatty acid oxygenase (AG1IA_01703)	-1.73	20-HETE (MEDP1153)	1.08	-0.952116108
		fatty acid oxygenase (AG1IA_01703)	-1.73	12(R)-HETE (MW0012180)	1.05	-0.967666938
		fatty acid oxygenase (AG1IA_01703)	-1.73	Trioxilin A3 (MW0102970)	1.08	-0.945032625
2	Ubiquinone and other terpenoid-quinone biosynthesis	fungal specific transcription factor domain-containing protein (AG1IA_02848)	1.07	6-Geranylgeranyl-2-methylbenzene-1,4-diol (MW0143834)	2.54	0.967221768
		NADH-quinone oxidoreductase (AG1IA_04313)	1.1	6-Geranylgeranyl-2-methylbenzene-1,4-diol (MW0143834)	2.54	0.926715638
3	Valine, leucine and isoleucine degradation	enoyl-CoA hydratase (AG1IA_02110)	1.04	3-Methyl-2-Oxovalerate (MEDL01886)	1.89	0.906403365
		alcohol dehydrogenase groES-like domain-containing protein (AG1IA_05942)	1.07	3-Methyl-2-Oxovalerate (MEDL01886)	1.89	0.958125942
		alcohol dehydrogenase groES-like domain-containing protein (AG1IA_05942)	1.07	3-Methyl-1-hydroxybutyl-ThPP (MW0119688)	15.02	0.969033244
4	Purine metabolism	adenosine deaminase (AG1IA_03187)	1.29	Adenosine 5'-Monophosphate (MEDN0153)	2.68	0.933350829
		adenosine deaminase (AG1IA_03187)	1.29	2'-Deoxyadenosine-5'-Monophosphate	1.99	0.959139816
		adenosine deaminase (AG1IA_03187)	1.29	3'-Aenylic Acid (MEDP0152)	2.42	0.949319684
		adenosine deaminase (AG1IA_03187)	1.29	Guanosine-5'-monophosphate (MEDP0169)	1.49	0.942176246
		adenosine deaminase (AG1IA_03187)	1.29	Cyclic Amp (MEDP0374)	1.63	0.965676531
		adenosine deaminase (AG1IA_03192)	-1.22	Xanthine (MEDN0140)	-1.07	0.855162935
		adenosine deaminase AG1IA_03192	-1.22	Adenine (MEDP0159)	-1.18	0.934916385
5	Tryptophan metabolism	NADPH--cytochrome P450 reductase (AG1IA_03629)	-1.7	2-Picolinic Acid (MEDN0098)	-1.86	0.858624279
		NADPH--cytochrome P450 reductase (AG1IA_03629)	-1.7	L-Tryptophan (MEDP0025)	-2.73	0.845399039
		NADPH--cytochrome P450 reductase (AG1IA_03634)	-1.67	2-Picolinic Acid (MEDN0098)	-1.86	0.95503802
		NADPH--cytochrome P450 reductase (AG1IA_03634)	-1.67	L-Tryptophan (MEDP0025)	-2.73	0.944983806
		aldehyde dehydrogenase (AG1IA_03762)	-1.11	2-Picolinic Acid (MEDN0098)	-1.86	0.814299365
		P450 family fatty acid hydroxylase (AG1IA_09807)	-1.08	2-Picolinic Acid (MEDN0098)	-1.86	0.863771777
		P450 family fatty acid hydroxylase (AG1IA_09807)	-1.08	L-Tryptophan (MEDP0025)	-2.73	0.856930949

		cytochrome P450 domain-containing protein(AG1IA 10228)	-1.76	2-Picolinic Acid (MEDN0098)	-1.86	0.962184225
		cytochrome P450 domain-containing protein(AG1IA 10228)	-1.76	L-Tryptophan (MEDP0025)	-2.73	0.958859357
6	Amino sugar and nucleotide sugar metabolism	glucose-6-phosphate isomerase (AG1IA 02400	1.02	Uridine-5'-diphospho-N-acetylgalactosamine disodium salt (MEDN1117)	1.16	0.965454044
		glucose-6-phosphate isomerase	1.02	Guanosine diphosphate mannose (MW0103583)	2.72	0.970171816
		carbohydrate esterase family 4 protein (AG1IA 08712)	2.56	Uridine-5'-diphospho-N-acetylgalactosamine disodium salt (MEDN1117)	1.16	0.932738438
		carbohydrate esterase family 4 protein (AG1IA 08712)	2.56	Guanosine diphosphate mannose (MW0103583)	2.72	0.954204629
		glycosyltransferase family 2 protein (AG1IA 09260)	-1.35	Uridine-5'-diphospho-N-acetylgalactosamine disodium salt (MEDN1117)	1.16	-0.974632196
		glycosyltransferase family 2 protein (AG1IA 09260)	-1.35	Guanosine diphosphate mannose (MW0103583)	2.72	-0.932468515
